# Astrocyte-derived exosomal nicotinamide phosphoribosyltransferase (Nampt) ameliorates ischemic stroke injury by targeting AMPK/mTOR signaling to induce autophagy

**DOI:** 10.1038/s41419-022-05454-9

**Published:** 2022-12-20

**Authors:** Yang Deng, Rui Duan, Wangli Ding, Qiuchen Gu, Manman Liu, Junshan Zhou, Jianguo Sun, Junrong Zhu

**Affiliations:** 1 Department of Pharmacy, Nanjing First Hospital, Nanjing Medical University, 210006 Nanjing, China; 2 School of Basic Medicine & Clinical Pharmacy, China Pharmaceutical University, 211198 Nanjing, China; 3 Department of Neurology, Nanjing First Hospital, Nanjing Medical University, 210006 Nanjing, China; 4 Key Lab of Drug Metabolism and Pharmacokinetics, State Key Laboratory of Natural Medicines, China Pharmaceutical University, 210009 Nanjing, China

**Keywords:** Cellular neuroscience, Cell death in the nervous system, Stroke

## Abstract

Acute ischemic stroke (AIS) is a global cerebrovascular disease with high disability and mortality, which has no effective therapy. Studies have demonstrated that astrocyte-derived exosomes (ADEXs) provided neuroprotection in experimental stroke models. Nevertheless, the role of exosomes derived from oxygen-glucose-deprivation/reoxygenation-stimulated astrocytes (OGD/R-stimulated astrocytes; OGD/R-ADEXs) in AIS remains largely unknown. Here, we found that OGD/R-ADEXs significantly reduced OGD/R-induced neuronal death and promoted neuronal autophagy. These effects were reversed when astrocytes were pretreated with GW4869, an exosome secretion inhibitor, or when hepatocyte growth factor-regulated tyrosine kinase substrate (Hrs) was knocked down. Neuroprotection was also observed during treatment with OGD/R-ADEXs in vivo. Further studies showed that Nampt, played a vital effect in the regulation of autophagy, was significantly increased in OGD/R-ADEXs. Knockdown of Nampt in astrocytes abolished the above-mentioned effects of OGD/R-ADEXs. Mechanistically, Nampt increased autophagy and decreased cell death by modulating AMPK/mTOR signaling, which recognized as a key signaling pathway of autophagy after AIS. Collectively, these results showed that Nampt released by OGD/R-ADEXs ameliorated acute ischemic stroke during neuronal injury by targeting AMPK/mTOR signaling to induce autophagy. Our study revealed a new key factor in the secretion of exosomes by OGD/R astrocytes, which regulated autophagy and induced neuroprotection in a mouse stroke model.

## Introduction

Acute ischemic stroke (AIS) is defined as a sudden decrease in blood flow to regions of the brain, resulting in a loss of neurological function [1]. In the past 20 years, although the treatment of acute cerebral ischemia has made great progress, intravenous administration of recombinant tissue plasminogen activator is still the main treatment for this disorder [2, 3]. It is therefore imperative to identify the underlying molecular mechanisms involving AIS and to develop new therapeutic approaches. In order to develop effective therapeutic methods and targets, it is necessary to determine whether the treatment is beneficial to multiple cell types. Among the multiple cell types that could be targeted, acting on astrocytes is particularly promising in the possible treatment of AIS [4]. Numerous studies recently have reported that regulation of astrocytes can alleviate the injury of ischemic stroke. Moreover, astrocytes may regulate transmission of synapses, and also have the potential to block and restore synaptic dysfunction after ischemic stroke [5]. Furthermore, induced neurons converted by reactive astrocytes can repair and recover damaged neuronal functions after ischemic stroke [6]. Overall, these studies suggested that targeting astrocytes can be a possible treatment for AIS. Thus, it is urgent to identify the underlying mechanism of astrocyte protection during AIS.

Exosomes are small vesicles with diameters of 50–140 nm, which can carry proteins, mRNA, and miRNA to communicate between cells [7]. As new carriers of signal transmission, astrocyte-derived exosomes (ADEXs) have been found to play crucial roles in many diseases, such as Alzheimer’s disease [8], hypoxic-ischemic brain damage [9], traumatic brain injury [10], Parkinson’s disease [11], amyotrophic lateral sclerosis [12], spinal cord injuries [13], Huntington’s disease [14], and ischemic stroke [15, 16]. Although ADEXs have been shown to improve the pathology of ischemic stroke, whether exosome-derived from oxygen-glucose-deprivation/reoxygenation (OGD/R)-stimulated ADEXs exert a protective role in AIS and its mechanism of action remain to be fully characterized.

Nicotinamide phosphoribosyltransferase (Nampt, also known as pre-B cell colony enhancing factor or visfatin), is a secreted protein that plays a pivotal role during cerebral ischemia injury [17]. For example, inhibition of Nampt aggravates cerebral infarction in rats with experimental cerebral ischemia, while overexpression of Nampt decreases ischemia-induced cerebral injuries [18]. Nevertheless, no data are available regarding the biological roles of exosomal Nampt of astrocytes in AIS.

A recent work has highlighted autophagy as a key factor in ischemic stroke [19]. Notably, autophagy acts as a double-edged sword in neuronal survival during cerebral ischemia [20]. Moderate induction of autophagy during cerebral ischemia is associated with increased cell viability and reduced infarct size [21–24]. However, prolonged oxygen and glucose deprivation or ischemia drives excessive autophagy, transforming temporarily activated and protective autophagy into chronically activated autophagy, which in turn leads to significant cell death [25–28]. Taken together, these studies indicate that autophagy has both positive and harmful effects, which may be caused by differences in the nature and time of cerebral ischemia. However, it is not clear whether oxygen-glucose-deprivation/reoxygenation (OGD/R)-ADEXs protect neurons by regulating autophagy after AIS.

In the present study, we characterized the role and mechanism of exosomes in astrocyte-mediated neuroprotection in models of AIS, both in vivo and in vitro. The results sugguested that Nampt released by OGD/R-ADEXs targeted the autophagic signaling pathway to activate AMPK and inhibit the phosphorylation of mTOR, thereby promoting neuronal autophagy, leading to increases in neuronal survival after induction of AIS. Overall, our results identified a novel pathway by which Nampt regulates neuronal autophagy, providing a potentially effective therapeutic target for AIS patients.

## Materials and methods

### Primary astrocytes culture

Primary astrocytes were isolated from the cerebral cortex of neonatal mice (1–3 days old). The chopped cortical tissue was digested using 0.125% trypsin (25200-056, Gibco, New York, USA), and the digest was filtered and centrifuged, then incubated in DMEM/F12 (11320033, Gibco, New York, USA) complete medium. After 9–11 days, the cells were shaken at 260 rpm at 37 °C for 16 h to remove the microglia and oligodendrocytes. Then, the purified astrocytes were cultured in DMEM/F12 supplemented with 10% fetal bovine serum (10100147, Gibco, New York, USA) and 1% penicillin/streptomycin (15140122, Gibco, New York, USA) in a 37 °C and 5% CO_2_ humidified incubator. The isolated astrocytes were characterized by staining marker GFAP (1:500; 3670, Cell Signaling Technology, Boston, USA).

### Primary neurons culture

Primary neurons were extracted from the cerebral cortex of fetal mice (16–18-days old). In brief, the cells were digested with 0.125% trypsin and grown with DMEM in culture flasks pretreated with poly-d-lysine (PDL) (A-003-M, Sigma-Aldrich, MO, USA), then the DMEM was replaced with neurobasal medium containing 2% B27 (17504044, Gibco, USA), and 0.5 mmol/L glutamine(G7513, Sigma-Aldrich, USA) at 5 h after seeding. The isolated neurons were characterized by neuronal marker MAP2 (1:500; ab11268, Abcam, Cambridge, UK). When the confluency reached 70–90%, the cells were used for further experiments.

### Cell transfection

LV-Nampt, sh-Nampt, Hrs-siRNA, and their control lentivirus (RiboBio, Guangzhou, China) were transfected into primary astrocytes or neurons using Lipofectamine 3000 (Invitrogen, CA, USA) according to the manufacturer’s protocol. The cells transfected 24 h later were used for subsequent experiments. Supplementary Table S1 lists the siRNA and shRNA sequences.

### Exosome isolation and characterization

Differential centrifugation was used to extract exosomes [29] and all steps were performed at 4 °C. Astrocytes were cultured to 80–90% confluence in DMEM/F12 complete medium containing 10% fetal bovine serum and 1% penicillin/streptomycin. Then, the complete medium was replaced with serum-free medium (EXO-FBS-50A-1, Shanghai, China). Exosomes isolated from 24 bottles of T175 cell culture flasks were purified from the cell culture supernatant of astrocytes under two different conditions: 24 h under normoxic medium (ADEXs) or 2 h OGD followed by 24 h reoxygenation in normoxic medium (OGD/R-ADEXs). Then, the supernatant was collected and centrifugated at 300 and 2000 × *g* to remove cells and other debris, filtered by 0.22 μm filters (Millipore, MA, USA) and centrifugated at 10,000 × *g* for 70 min to precipitate exosomes. Exosomes were resolved in PBS by centrifugation at 10,000 × *g* for 70 min again and dissolved in PBS. Exosomes were used directly or stored at −80 °C. To visualize the morphology of exosomes, a Tecnai G2 Spirit Bio TWIN transmission electron microscope (FEI, Hillsboro, OR, USA) was used to observe exosomes. A NanoSight NS500 instrument (NanoSight Technology, Malvern, UK) was used to evaluate the size distribution of exosomes. Next, the levels of exosomal marker proteins, namely CD63, HSP70, and TSG101, were detected by western blotting. To assess intravesicular localization of Nampt, OGD/R-ADEXs were treated with 100 μg/mL proteinase K (70663, Sigma-Aldrich, USA) alone or with proteinase K and 0.5% Triton X-100 (X-100, Sigma-Aldrich, USA) at 37 °C for 60 min, then analyzed using western blotting [30].

### Detection of exosome uptake and inhibition

To monitor the uptake of exosomes, a PKH67 (green) kit (Sigma-Aldrich) was used to label the isolated exosomes following the manufacturer’s protocol. After incubating with neurons for 24 h, the neurons were fixed and observed using a TCS-SP2 confocal laser scanning microscope (Leica, Wetzlar, Germany). To detect the biological distribution of exosomes in vivo, mice were injected with the abovementioned, labeled OGD/R-ADEXs via the tail vein at reperfusion and sacrificed 2 h later. The brain slices were then counterstained with anti-NeuN (1:200; 66836-1-Ig, Proteintech, Wuhan, China) and 4’,6-diamidino-2-phenylindole (DAPI) and the biological distribution was observed using an immunofluorescence assay.

For experiments requiring exosomal inhibition by GW4869, the astrocytes were cultured to 80–90% confluency in DMEM/F12 complete medium supplemented with 10% FBS and 1% penicillin/streptomycin. 10 µM GW4869 (D1692, Sigma-Aldrich, USA) was added to the DMEM/F12 medium for 24 h before exosome isolation.

### The OGD/R model

Primary astrocytes underwent glucose and oxygen deprivation followed by reoxygenation to mimic the environment of cerebral ischemia in vitro. In short, astrocytes were cultured with DMEM (10566016, Gibco, New York, USA) without glucose and FBS (10100147, Gibco, New York, USA) in a 95% N_2_ and 5% CO_2_ chamber for 2 h. After hypoxia, the glucose-free DMEM medium was replaced with DMEM/F12 complete medium and cultured in a 95% air and 5% CO_2_ incubator for 24 h. Next, the astrocyte culture medium was collected and filtered through a 0.22 mm filter to remove dead cells and debris. In a similar manner, primary neurons were incubated in the hypoxia chamber for 2 h and then cultured in normal neural basal medium for different durations of time.

### Astrocytes-neuron co-culture and administration

Astrocytes were seeded on 6-well Transwell chambers (Costar, Maryland, USA) at a density of 2 × 10^4^ cells/well. After 24 h, primary neurons were seeded on the plates and co-cultured using the previous normoxia or hypoxia condition. Primary neurons in the OGD/R-ADEXs treatment groups were treated with three different concentrations of OGD/R-ADEXs and neurons in the 3-MA treatment groups were treated with 5 mM 3-MA (5142-23-4, Sigma-Aldrich, USA) dissolved in PBS. To aviod 3-MA not working due to its short half-life time, 3-MA were added to the neural basal medium twice, at the beginning of hypoxia and reoxygenation.

### Neuron viability measurement

Cell viability was measured using the CCK-8 assay as described herein. Briefly, primary neurons (3 000 cells/well) were plated in 96-well plates and treated with OGD/R astrocytes and OGD/R-ADEXs. Subsequently, 10 µL CCK-8 solution (96992, Sigma-Aldrich, USA) was added and incubated at 37 °C for 3 h. Finally, the optical density at 450 nm was measured using a microplate reader (Thermo Scientific, San Jose, CA, USA).

Neuronal death was measured by the release of lactate dehydrogenase (LDH) in the medium supernatant. In brief, neurons treated with OGD/R and transfected in 6-well plates were collected, suspended in a 96-well plate, and then incubated at 37 °C for 30 min. The absorbance of the cell supernatant at 490 nm was then measured using a microplate reader according to the instructions of the LDH assay kit (MAK066, Sigma-Aldrich, USA). The LDH activity was calculated as absorbance of the sample well – absorbance of the control well)/(absorbance of the standard – absorbance of the standard blank) using the concentration of the standard substance (0.2 μmol/mL) [31].

### Immunofluorescence assays of brain tissue and cells

Brain tissue was embedded in paraffin and cut into 4 µm slices. The slices were then deparaffinized by xylene and rehydrated with different concentrations of ethanol. The antigen was regenerated using sodium citrate buffer (pH 6.0) for 10 min. After blocking with 1% goat serum and PBS (containing 0.1% Triton X-100) at room temperature for 30 min, anti-LC3B (1:200; ab48394, Abcam,UK), anti-MAP2 (1:500; ab11268, Abcam,UK) and anti-NeuN (1:200; 66836-1-Ig, Proteintech, China) antibodies were added and incubated overnight at 4 °C. After washing with PBS three times, secondary antibody working solution was added and incubated at room temperature for 1 h. The slices were stained with DAPI for 5 min and observed using a light microscope (Zeiss, Oberkochen, Germany). For neurons, 4% paraformaldehyde was used to fix the cells, and 0.2% bovine serum albumin was used for blocking for 30 min. The subsequent anti-LC3B antibody staining steps were the same as those used for the brain tissues

### Detection of autophagosomes by electron microscopy

The ipsilateral cortex (six mice per group) and cell samples in vitro were fixed with 2.5% glutaraldehyde (G5882, Sigma-Aldrich, USA). Then, 0.2 M phosphate buffer (pH 7.2; Gibco, New York, USA) was used to wash the samples twice for 15 min, and then 1% osmium tetroxide (Sigma-Aldrich, USA) was used to fix the samples for 2 h. After dehydration in a gradient of ethanol, the samples were embedded with epoxy resin and sliced into thin sections. Finally, the sections were stained with uranyl acetate and lead citrate, and observed by TEM. In our experimental study, vesicles with double-membrane structures enveloping cytoplasmic material were defined as autophagosomes, which were quantified by the number of autophagosomes (Black arrowheads) per square micrometer.

### Western blotting

Total proteins were extracted from exosomes or primary neurons or mice brain tissues using RIPA lysis buffer (89900, Thermo Scientific, CA, USA) and quantified by the BCA protein assay kit (23225, Thermo Scientific, CA, USA). Equal amounts of protein samples (30–40 µg protein/lane) were separated by 12% sodium dodecyl-sulfate–polyacrylamide gel electrophoresis and then transferred to polyvinylidene difluoride (PVDF) membranes (Merck Group, Darmstadt, Germany). Next, the membranes were incubated with primary antibodies against LC3B (1:1000; ab48394, Abcam, Cambridge, UK), Nampt (1:5000; 11776-1-AP, Proteintech, China), AMPK (1:1000; 5831, Cell Signaling Technology, Boston, USA), mTOR (1:1000; 2983, Cell Signaling Technology, Boston, USA), p-AMPK (1:1000; AF3423, Affinity, Michigan, USA), p-mTOR (1:1000; AF3308, Affinity, Michigan, USA), and β-actin (1:1000; 4970, Cell Signaling Technology, Boston, USA) overnight at 4 °C after blocking with 5% nonfat milk for 1 h. After washing three times with TBST, the membranes were incubated with horseradish peroxidase-conjugated goat anti-rabbit IgG antibody (1:3000; 7074, Cell Signaling Technology, Boston, USA) at room temperature for 1 h. For exosomal marker proteins including CD63 (1:1000; ab217345, Abcam, Cambridge, UK), Tsg101 (1:1000; ab125011, Abcam, Cambridge, UK), Hsp70 (1:1000; ab181606, Abcam, Cambridge, UK), and negative control IDE (1:10000; ab109538, Abcam, Cambridge, UK) were separated and measured by the same procedures. Then, the membranes were washed with TBST three times again and visualized using an Enhanced Chemiluminescence Plus Kit (Thermo Fisher Scientific). Images were analyzed by software ImageJ (National Institutes of Health, Bethesda, Maryland, USA).

### Experimental animals

Adult male C57BL/6 mice (25–30 g, 8–10 weeks) were purchased from Vital River (Beijing, China) and raised in a room with temperature (22 ± 2 °C), humidity (50–60%) and 12 h light/dark cycle (six per cage) and given free access to food and water. All experiments were approved by the Ethics Committee of Nanjing First Hospital (approval number: DWSY-1801173) and were implemented according to the National Institute of Health Guide for the Care and Use of Laboratory Animals (NIH Publication no. 80-23, revised 1996). At least five mice were analyzed for each data point. All the mice were allocated to groups and processed with randomization and the investigator was blinded to the group allocation. Precise numbers of animals used are given for each condition in the figure legends and in the Supplementary Table S2 including survival rates of mice.

The first set of mice underwent MCAO followed by injection of PBS (MCAO) or OGD/R-ADEXs. OGD/R-ADEXs were injected intravenously through the tail vein into ischemic mice at a concentration of 20 μg/mL at the beginning of the reperfusion followed by a survival of 24 h (six mice per group). The brains were removed and used for infarct volume analysis as described later.

The second set of mice was subjected to 45 min of ischemia followed by 6, 12 or 24 h of reperfusion (six mice per group). All these groups were also used for western blot analysis.

The third set of mice was exposed to MCAO followed by administration of PBS (MCAO) or OGD/R-ADEXs immediately at the beginning of the reperfusion. The mice were allowed to survive for 1, 3, 7, or 14 days (six mice per group). These mice were used for Behavioral test, Brain water content, Immunohistochemistry, Immunofluorescence, Transmission electron microscopy and western blot analysis of ischemic injury.

The fourth set of mice was exposed to MCAO followed by administration of PBS (MCAO), OGD/R-ADEXs, OGD/R-ADEXs^sh-NC^ or OGD/R-ADEXs^sh-Nampt^ immediately at the beginning of the reperfusion. The mice were allowed to to survive for 24 h post-stroke (six mice per group). These mice were used for Behavioral test, Brain water content, Immunohistochemistry, Immunofluorescence, Transmission electron microscopy and western blot analysis of ischemic injury.

### MCAO/R model

Briefly, mice were anesthetized by 2% isoflurane in O_2_ (RWD Life Science, Shenzhen, China). A midline neck incision was made to expose the right internal carotid artery (ICA), external carotid artery (ECA), and common carotid artery. After ligating the ECA, silicon-coated monofilament (diameter: 0.18 ± 0.01 mm; Cinontech, Beijing, China) was inserted from the ECA into the ICA until slight resistance was felt. Then, the monofilament was withdrawn after 45 min occlusion to induce reperfusion. To verify that MCA was accurately occluded, Perfusion Speckle Image System (FLP12; Gene & I, Beijing, China) was used to monitor changes of regional cerebral blood flow (rCBF) during and after surgery. A decrease in rCBF ≥ 75% of baseline was considered as successful occlusion of MCA. During the surgery, the body temperature of the mouse was kept at 37 ± 0.5 °C using a heating pad. Sham-operated mice underwent the same procedure, except that the monofilament was not inserted.

### TTC staining

Triphenyl tetrazolium chloride (TTC) staining was performed at 24 h after surgery. Firstly, the brains of mice were cut into six 2-mm thick sections starting from the frontal region. Then, sections were stained using 2% TTC (17779, Sigma-Aldrich, USA) solution at 37 °C for 30 min, and fixed with 4% paraformaldehyde. ImageJ software (National Institutes of Health) was used to calculate the percentages of normal tissue (dark red regions) and infarcted areas (white regions).

### Neurological severity score and behavioral testing

The neurological deficits of mice were assessed following the method described by Longa et al. [32] at 1, 3, 7 or 14 days after surgery, by an investigator who was blinded to the experimental groups. The Longa score was assessed as follows: 0, no neurological deficit, normal walking; 1, mild neurological deficit, failure to fully extend contralateral forepaw; 2, moderate neurological deficit, circling to the contralateral side; 3, severe neurological deficit, falling to the contralateral side; 4, no spontaneous moving with depressed consciousness level. Mice with a score of 0 or 4 were eliminated from this study. Behavioral tests including the rotarod and corner tests were performed before surgery and 1, 3, 7 or 14 days after surgery. Again, all behavioral tests were performed by investigators who were blinded to the identities of the groups.

### Detection of cerebral edema

The standard wet-dry method was used to detect brain water content to assess cerebral edema. After removal, the wet weighs of brains (wet weight) were immediately determined, and the brains were then dried at 100 °C for 24 h (dry weight). The brain edema = (wet weight – dry weight)/wet weight × 100% [33].

### Histopathology examination

#### HE staining

At 24 h after MCAO/R, mice were deeply anesthetized with 2% isoflurane in O_2_, and then normal saline (200 mL) and 4% paraformaldehyde (200 mL) were perfused into the heart. The brains were then removed and cut into sections (5 μm thick) and stained with HE. Histological changes were then observed using a CX23 microscope (Olympus, Tokyo, Japan).

#### Nissl staining

At 24 h after surgery, brain tissues were converted into paraffin sections. The sections were then rinsed with PBS and dyed with 1% toluidine blue for 40 min at 60 °C (or with tar violet for 30 s). The sections were then dehydrated in gradient ethanol, rinsed with xylene, and sealed with neutral gum after removing the dye by washing. The sections were then observed using the CX23 microscope (Olympus).

### Immunohistochemistry

The animals were anesthetized at 24 h after surgery, and the brains were fixed using left ventricle perfusion with normal saline, then incubation in 4% paraformaldehyde. The sections were deparaffinized by xylene, rehydrated with different concentrations of ethanol solutions, and treated with antigen regeneration using 10 mM sodium citrate buffer (pH 6.0). After blocked with normal goat serum at 37 °C for 30 min, the sections were then incubated with primary antibodies as follows by overnight incubation at 4 °C: Nampt (1:50; 11776-1-AP, Proteintech, China), p-AMPK (1:100; AF3423, Affinity, USA), and p-mTOR (1:100; AF3308, Affinity, USA) antibodies. Then, the sections were incubated with corresponding biotinylated secondary antibody at 37 °C for 30 min. Positive staining was determined after treatment with 3-3′diaminobenzidine (D8001, Sigma-Aldrich, USA). Finally, the sections were observed using a DM600B automatic microscope (Leica Microsystems, Heidelberg, Germany) and photographed, and then quantified by ImageJ software (National Institutes of Health).

### Statistical analysis

Prism 6 software (GraphPad, San Diego, CA, USA) was used for all statistical analyses. Data are presented as the mean ± standard deviation (SD) based on three independent experiments. All data were normally distributed, and variances between the groups that were being statistically compared were similar. Differences between groups were determined using the Student’s *t*-tests (two groups) and one-way ANOVA (no less three groups)followed by Tukey’s post hoc test. Neurological deficit score and behavioral test were analyzed by two-way repeated-measures ANOVA followed by Tukey’s post hoc test. A value of *P* < 0.05 was considered to be statistically significant.

## Results

### OGD/R-ADEXs protect neurons from oxygen-glucose-deprivation/reoxygenation (OGD/R) injury

**Fig. 1:**
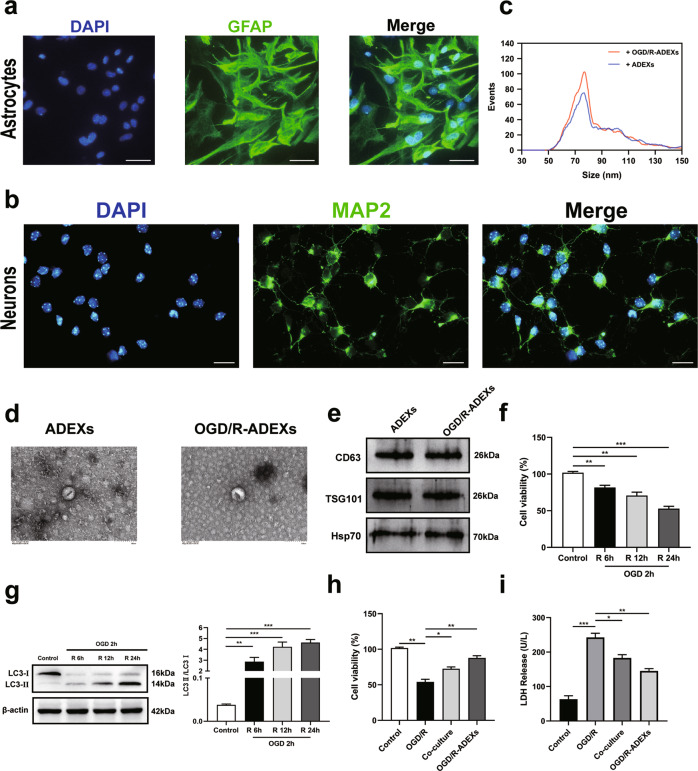
OGD/R-ADEXs protects neurons against oxygen-glucose deprivation (OGD)/reoxygenation (R). **a** Images of immunofluorescence of primary astrocytes. Scale bar: 20 μm. **b** Images of immunofluorescence of primary neurons. Scale bar: 20 μm. **c** NTA from enriched exosomes depicting size distribution patterns. **d** Representative images of exosomes from astrocytes. Scale bar: 100 nm. **e** The expression of CD63, TSG101, and Hsp70 were determined by western blotting. **f** Cell viability was detected in neurons exposed to OGD for 2 h followed by 6, 12, or 24 h of reoxygenation using the CCK-8 assay (*n* = 5). **g** The expressions of LC3 II/I were determined by western blotting using the previously mentioned time points (*n* = 5). **h** The neuroprotective effects of co-culture and OGD/R-ADEXs of neurons were evaluated using the CCK-8 assay (*n* = 5). **i** The neuroprotective effect of co-culture and OGD/R-ADEXs of neurons using the LDH assay (*n* = 5). Data are shown as the mean ± SD of at least three independent experiments. ^*^P < 0.05; ^**^P < 0.01; ^***^P < 0.001, Cells incubated under standard cell culture conditions (Control) were used as negative control; Cells treated with PBS under OGD/R conditions served as positive control (OGD/R); ADEXs, exosomes isolated from astrocytes; OGD/R-ADEXs, exosomes isolated from OGD/R astrocytes; Co-culture, OGD/R neurons co-cultured with OGD/R astrocytes.

To determine whether OGD/R-ADEXs had a protective effect on neurons, we first isolated primary astrocytes and neurons from C57/BL6 mice. As shown in Fig. 1a, b, immunofluorescence assay found that cells isolated in this article were consistent with the definition of astrocytes and neurons. We then enriched exosomes from the conditioned medium of astrocytes. Nanoparticle tracking analysis (NTA) showed that exosomes secreted from astrocytes in both groups were usually approximately 85 nm in size (Fig. 1c). In addition, transmission electron microscopy (TEM) results indicated that exosomes were present in the medium harvested from non-OGD/R astrocytes and OGD/R astrocytes (Fig. 1d). Consistent with previous reports [34], western blotting of exosome surface markers revealed the abundance of CD63, TSG101, and Hsp70 in these exosomes released from primary astrocytes (Fig. 1e). Hence, these isolated vesicles were considered to be ADEXs and OGD/R-ADEXs.

To further investigate the protective actions of OGD/R-ADEXs, neurons were co-cultured with OGD/R astrocytes (Co-culture) or OGD/R-ADEXs. As expected, neurons exposed to OGD and then followed by 24 h reoxygenation showed remarkable cell damage, depending on the induction time of the OGD (Fig. 1f). Moreover, the autophagy levels were detected using western blotting. Microtubule-associated protein light chain 3 (LC3 II/I) expressions, used to monitor autophagy, were significantly up-regulated in neurons exposed to OGD for 2 h followed by 24 h reoxygenation, when compared with 6 h or 12 h reoxygenation (Fig. 1g). In this study, OGD for 2 h followed by 24 h reoxygenation were therefore used for further in vitro experiments. Treatment of neurons exposed to OGD/R with OGD/R astrocytes or with OGD/R-ADEXs significantly reduced cell injury (Fig. 1h, i). Collectively, these results showed that OGD/R-ADEXs alleviated OGD/R-induced neuronal injury.

### OGD/R-ADEXs protect neurons from OGD/R injury by regulating autophagy

**Fig. 2:**
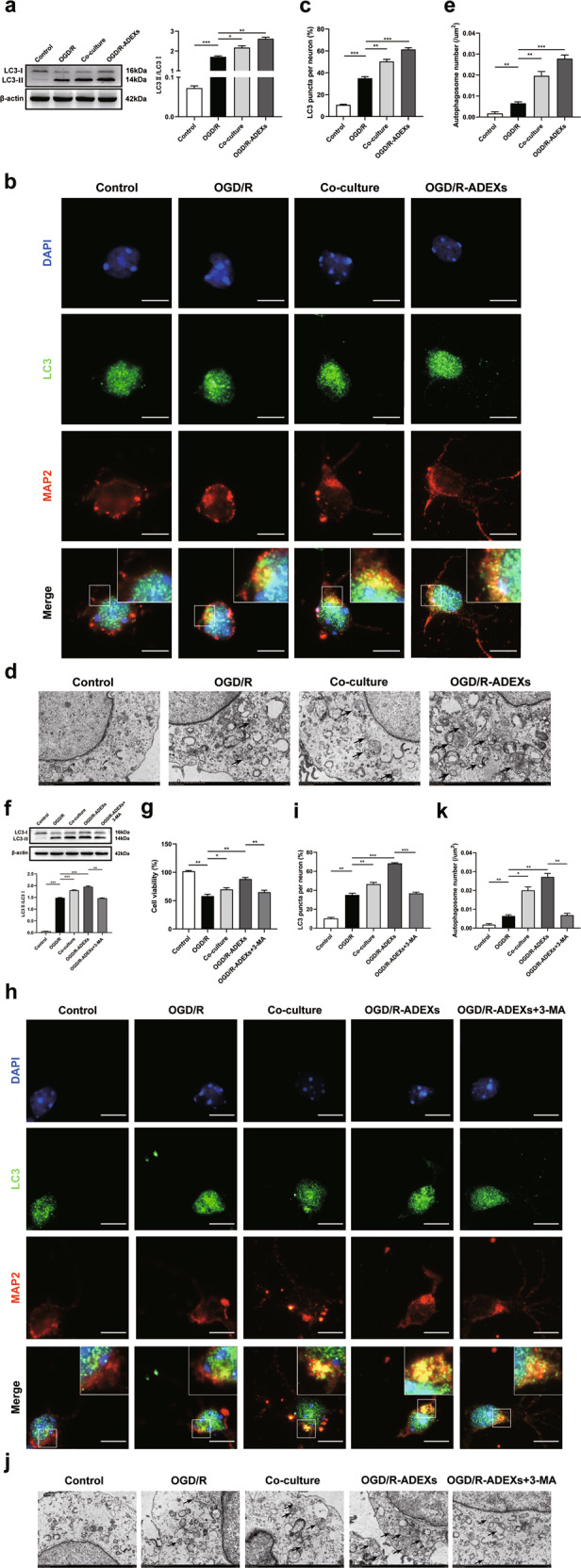
OGD/R-ADEXs protect neurons against oxygen-glucose deprivation/reoxygenation (OGD/R) injury through regulating autophagy. **a** The expressions of LC3 II/I were determined by western blotting in OGD/R-induced neurons treated with OGD/R astrocytes or OGD/R-ADEXs (*n* = 5). **b**, **c** Representative photomicrographs and quantification of LC3-positive puncta in OGD/R-induced neurons treated with OGD/R astrocytes or OGD/R-ADEXs. Scale bar: 10 μm (*n* = 5). **d**, **e** The autophagosomes in OGD/R-induced neurons treated with OGD/R astrocytes or OGD/R-ADEXs were detected by transmission electron microscopy. Scale bar, 1 μm (*n* = 5). **f** The expressions of LC3 II/I were determined by western blotting after OGD/R-ADEXs were used together with 3-methyladenine (3-MA) (*n* = 5). **g** The effects of autophagy on neuronal survival were assessed using the CCK-8 assay after OGD/R-ADEXs were used, together with 3-MA (*n* = 5). **h**, **i** Representative photomicrographs and quantitation of LC3-positive puncta in OGD/R-induced neurons after OGD/R-ADEXs were used together with 3-MA (*n* = 5). Scale bar: 10 μm. **j**, **k** Autophagosomes in OGD/R-induced neurons were detected by transmission electron microscopy after OGD/R-ADEXs were used together with 3-MA (*n* = 5). Scale bar, 1 μm. Data are shown as the mean ± SD of at least three independent experiments. ^*^P < 0.05; ^**^P < 0.01; ^***^P < 0.001. Neurons incubated under standard cell culture conditions (Control) were used as negative control; Neurons treated with PBS under OGD/R conditions served as positive control (OGD/R); OGD/R-ADEXs, exosomes isolated from OGD/R astrocytes; Co-culture, OGD/R neurons co-cultured with OGD/R astrocytes; OGD/R-ADEXs+3-MA, OGD/R-ADEXs used together with 5 mM 3-MA.

To determine whether OGD/R-ADEXs enhanced neuronal resistance against OGD/R by regulating autophagic activity, the expressions of LC3 II/I, autophagy plaques, and autophagosomes were used to assess the levels of autophagy. When neurons were exposed to OGD/R treated with OGD/R astrocytes or OGD/R-ADEXs, the increased LC3 II/I protein expressions induced by stroke were further enhanced (Fig. 2a). Immunofluorescence analyses consistently showed that OGD/R treatment considerably promoted the production of neuronal autophagy plaques, which was induced by OGD/R astrocytes and OGD/R-ADEXs (Fig. 2b, c). Furthermore, we also used TEM to monitor the autophagosomes. Primary neurons exposed to OGD/R incubated with OGD/R astrocytes or OGD/R-ADEXs strongly promoted the formation of autophagosomes (Fig. 2d, e). More importantly, administration of 3-methyladenine (3-MA), a known autophagy inhibitor, reversed the increases in LC3 II/I levels (Fig. 2f) and in cell viability (Fig. 2g) of neurons induced by OGD/R astrocytes and OGD/R-ADEXs. The autophagy plaques and autophagosome also showed similar results (Fig. 2h–k).

To verify the physiological characteristics of OGD/R-ADEXs in this OGD/R model, PKH67 staining was used to determine exosome uptake in neurons. As expected, primary neurons showed intracellular uptake of exosomes after OGD/R (Supplementary Fig. 1a). We also wondered whether the effect induced by OGD/R-ADEXs was dose-dependent. Three different doses (10 μg/mL, 20 μg/mL, and 30 μg/mL) of OGD/R-ADEXs were therefore used in the OGD/R model. OGD/R-ADEXs remarkably enhanced LC3 II/I expressions (Supplementary Fig. 1b) and decreased cell damage (Supplementary Fig. 1c, d) in a dose-dependent manner. Furthermore, a moderate dosage of OGD/R-ADEXs (20 μg/mL) displayed beneficial effects on cell survival. Thus, this dosage was selected for further experiments.

Additionlly, we measured the cell survival of neurons co-cultured with OGD/R-ADEXs that were pretreated with either Protease K, Triton X-100, both, or with untreated OGD/R-ADEXs after OGD/R. When OGD/R-ADEXs were pretreated with Triton X-100, but not with Protease K, and co-cultured with primary neurons, there was no significant difference in cell viability after OGD/R between Triton X-100 treatment and the OGD/R-ADEXs group. Pretreatment of OGD/R-ADEXs with Protease K alone partially decreased the neuroprotective effect. In contrast, pretreatment of OGD/R-ADEXs with Triton X-100 and Protease K resulted in almost complete elimination of the OGD/R-ADEXs protective properties (Supplementary Fig. 1e). Together, these results suggested that OGD/R-ADEXs were an effective therapeutic treatment, when use in the OGD/R model.

### Inhibition of exosome biogenesis reverses the effects of OGD/R-ADEXs in neurons exposed to OGD/R

**Fig. 3:**
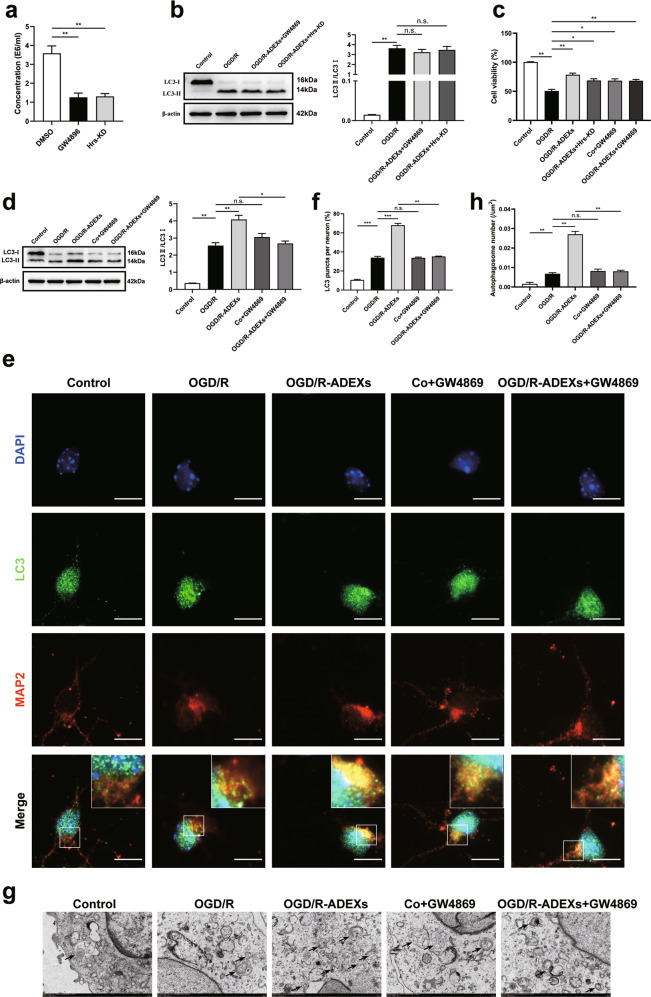
The regulation of autophagy by OGD/R-ADEXs depends on exosomes. **a** The quantitation of OGD/R-ADEXs obtained from OGD/R astrocytes pretreated with dimethyl sulfoxide (DMSO), GW4869, or Hrs-siRNA (knockdown, Hrs-KD) *(n* = 5). **b** The expression of LC3 II/I was determined by western blotting in OGD/R-induced neurons treated with PBS, OGD/R-ADEXs isolated from OGD/R astrocytes pretreated with GW4869 (OGD/R-ADEXs+GW4869) or pre-transfected with Hrs-KD (OGD/R-ADEXs+Hrs-KD) (*n* = 5). **c** The cell viability was detected in OGD/R-induced neurons treated with PBS, OGD/R-ADEXs, Co+GW4869, OGD/R-ADEXs+Hrs-KD, or OGD/R-ADEXs+GW4869 using the CCK-8 assay (*n* = 5). **d** The expressions of LC3 II/I were determined using western blotting of OGD/R-induced neurons treated with PBS, OGD/R-ADEXs, Co+GW4869, or OGD/R-ADEXs+GW4869 (*n* = 5). **e**, **f** Representative photomicrographs and quantitation of LC3-positive puncta in OGD/R exposed neurons treated with PBS, OGD/R-ADEXs, Co+GW4869, or OGD/R-ADEXs+GW4869 (*n* = 5). Scale bar: 10 μm. **g**, **h** Autophagosomes were detected by transmission electron microscopy of OGD/R-induced neurons treated with PBS, OGD/R-ADEXs, Co + GW4869, or OGD/R-ADEXs+GW4869 (*n* = 5). Scale bar: 1 μm. Data are shown as the mean ± SD of at least three independent experiments. ^*^P < 0.05; ^**^P < 0.01; ^***^P < 0.001. Neurons incubated under standard cell culture conditions (Control) were used as negative control; Neurons treated with PBS under OGD/R conditions served as positive control (OGD/R); OGD/R-ADEXs, exosomes isolated from OGD/R astrocytes; Co+GW4869, OGD/R astrocytes treated with the exosome secretion inhibitor GW4869; OGD/R-ADEXs+GW4869, OGD/R-ADEXs isolated from OGD/R astrocytes pre‐treated with GW4869; OGD/R-ADEXs+Hrs-KD, OGD/R-ADEXs isolated from OGD/R astrocytes pre-transfected with Hrs-KD; ns, not significant.

To confirm the regulatory effects of exosomes in autophagy and neuroprotection in OGD/R-induced neurons, GW4869, an inhibitor of exosomes secretion, was used to reduce exosome release and Hrs, a member of the ESCRT‐0 complex [35], was knocked down to inhibit ESCRT-mediated exosome biogenesis. As expected, both Hrs knockdown and GW4869 preconditioning resulted in a significant reduction in exosome release, as shown by nanoparticle tracking analysis (Fig. 3a), indicating that there was no difference between them. Additionally, when OGD/R astrocytes were pretreated with GW4869 or Hrs knockdown, the effects of OGD/R-ADEXs on autophagy regulation (Fig. 3b) and neuroprotection (Fig. 3c) were blocked. Subsequently, GW4869 was used in a co-culture model, showing that the effects of OGD/R-ADEXs on neuroprotection (Fig. 3c) and autophagy regulation (Fig. 3d–h) were blocked. Based on these findings, OGD/R-ADEXs appeared to be important in mediating autophagy regulation in OGD/R-stimulated neurons.

### Nampt is abundant in OGD/R-ADEXs, and promotes autophagy in hypoxic neurons

**Fig. 4:**
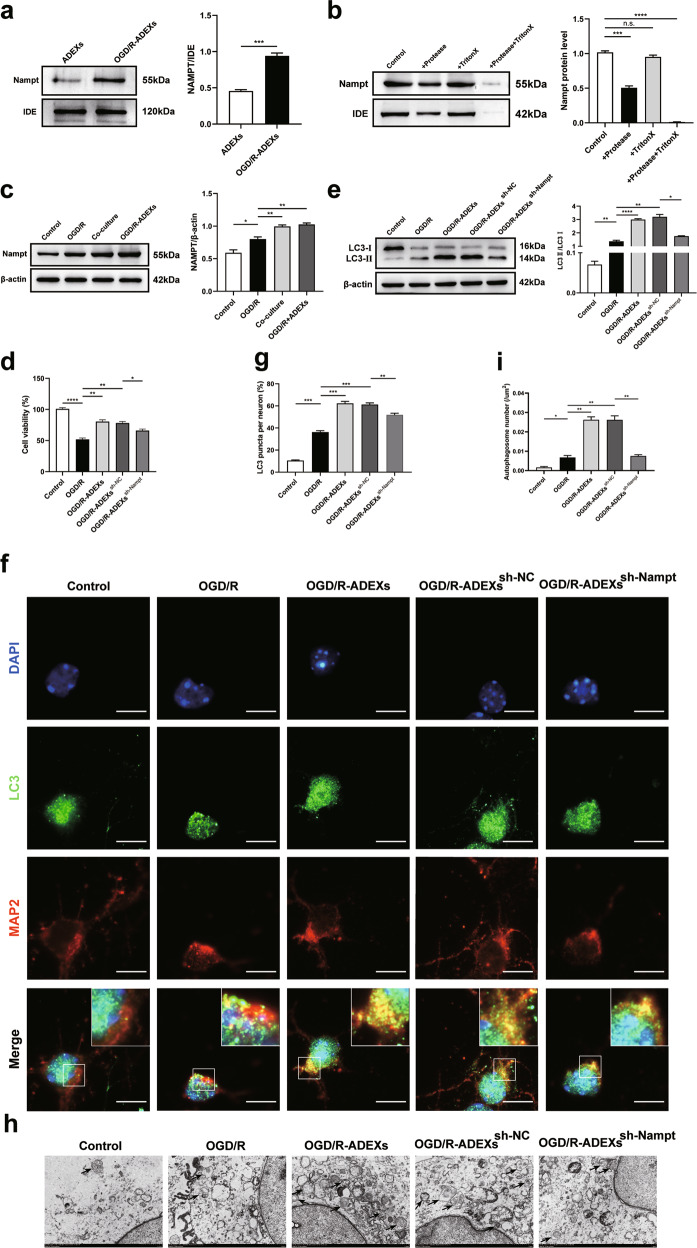
OGD/R-ADEXs regulate autophagy and provide neuroprotection using Nampt. **a** The expression of Nampt in exosomes obtained from astrocytes was determined by western blotting. IDE was, internal marker for exosomes, used as negative markers (*n* = 5). **b** The expression of Nampt in OGD/R-ADEXs that were pretreated with either Protease K (+Protease), the Triton X-100 detergent (+Triton X), both of them (+Protease+Triton X), or with the solvent alone (Control) was determined by western blotting, which was normalized to IDE (*n* = 5). **c** The expressions of Nampt in neurons treated with OGD/R astrocytes or OGD/R-ADEXs were determined by western blotting (*n* = 5). **d** The cell viability was detected in OGD/R-induced neurons treated with PBS, OGD/R-ADEXs, OGD/R-ADEXs^sh-Nampt^, or OGD/R-ADEXs^sh-NC^ using the CCK-8 assay (*n* = 5). **e** The expressions of LC3 II/I were determined by western blotting of OGD/R-induced neurons treated with PBS, OGD/R-ADEXs, OGD/R-ADEXs^sh-Nampt^, or OGD/R-ADEXs^sh-NC^. **f**, **g** Representative photomicrographs and quantitation of LC3-positive puncta in OGD/R-induced neurons treated with PBS, OGD/R-ADEXs, OGD/R-ADEXs^sh-Nampt^, or OGD/R-ADEXs^sh-NC^ (*n* = 5). Scale bar: 10 μm. **h, i** The autophagosomes were detected by transmission electron microscopy of OGD/R-induced neurons treated with PBS, OGD/R-ADEXs, OGD/R-ADEXs^sh-Nampt^, or OGD/R-ADEXs^sh-NC^ (*n* = 5). Scale bar: 1 μm. Data are shown as the mean ± SD of at least three independent experiments. ^*^P < 0.05; ^**^P < 0.01; ^***^P < 0.001; ^****^P < 0.0001. Neurons incubated under standard cell culture conditions (Control) were used as negative control; Neurons treated with PBS under OGD/R conditions served as positive control (OGD/R); ADEXs, exosomes isolated from astrocytes; OGD/R-ADEXs, exosomes isolated from OGD/R astrocytes; Co-culture, OGD/R neurons co-cultured with OGD/R astrocytes; OGD/R-ADEXs^sh-Nampt^, exosomes isolated from OGD/R astrocytes that were pretreated with sh-Nampt; OGD/R-ADEXs^sh-NC^, exosomes isolated from OGD/R astrocytes that were pretreated with scramble.

Lu et al. [36] recently reported that microglia actively secreted Nampt via exosomes during inflammation after cerebral ischemia. To further study whether the Nampt of exosomes was involved in the regulation of autophagy induced by OGD/R-ADEXs, we determined whether there were differences in the expressions of Nampt in ADEXs and OGD/R-ADEXs. The western blot results indicated that Nampt in the OGD/R-ADEXs was significantly increased, when compared with the ADEXs (Fig. 4a). To determine the physicochemical state of Nampt, the detergent, Triton X-100 and/or Protease K or solvent alone were used to treat exosomes. The protein expression of Nampt was then measured by western blotting. Exosomes treated with Protease K and Triton X-100 led to almost complete digestion of proteins (Fig. 4b), showing that their initial resistance to digestion resulted from isolation within vesicles, and demonstrating that Nampt was located inside the OGD/R-ADEXs. Next, we found that the intracellular concentration of Nampt in OGD/R-induced neurons increased. Consistently, treatment of neurons with OGD/R-ADEXs further increased the intracellular concentration of Nampt (Fig. 4c).

To verify whether Nampt mediated the role of the aforementioned exosomes in OGD/R-induced neuronal autophagy, exosomes were collected from OGD/R astrocytes, which were pretreated with a sh-Nampt (OGD/R-ADEXs^sh-Nampt^) or with a sh-NC (OGD/R-ADEXs^sh-NC^). Compared with PBS-treated controls, the cell viability after OGD/R was remarkably higher in neurons treated with OGD/R-ADEXs^sh-NC^. However, this effect was reversed when the cells were treated with OGD/R-ADEXs^sh-Nampt^ (Fig. 4d). The LC3‐II/I levels induced by OGD/R were also higher in neurons treated with OGD/R-ADEXs^sh-NC^, when compared with cells treated with PBS, while this result was decreased when neurons were incubated with OGD/R-ADEXs^sh-Nampt^ (Fig. 4e). Furthermore, using an immunofluorescence assay, the number of autophagy plaques significantly decreased in neurons incubated with OGD/R-ADEXs^sh-Nampt^, compared with OGD/R-ADEXs^sh-NC^ (Fig. 4f, g). In a similar manner, neurons incubated with OGD/R-ADEXs^sh-Nampt^ had less autophagosomes, when compared with OGD/R-ADEXs^sh-NC^ (Fig. 4h, i). We then used LV-Nampt and sh-Nampt to directly treat OGD/R-induced neurons to further analyze the effects of Nampt on autophagy regulation. Neurons transfected with LV-Nampt after OGD/R, enhanced autophagy regulation (Supplementary Fig. 2a), when compared with the scramble group. In contrast, transfecting neurons with sh-Nampt resulted in the opposite results (Supplementary Fig. 2c). In addition, transfection of OGD/R-stimulated neurons with LV-Nampt led to increased cell viability (Supplementary Fig. 2b), while sh-Nampt decreased neuron viability (Supplementary Fig. 2d). Together, these results indicated that Nampt was essential for regulating autophagy induced by OGD/R-ADEXs.

### OGD/R-ADEXs regulate autophagy by the AMPK/mTOR signaling pathway

**Fig. 5:**
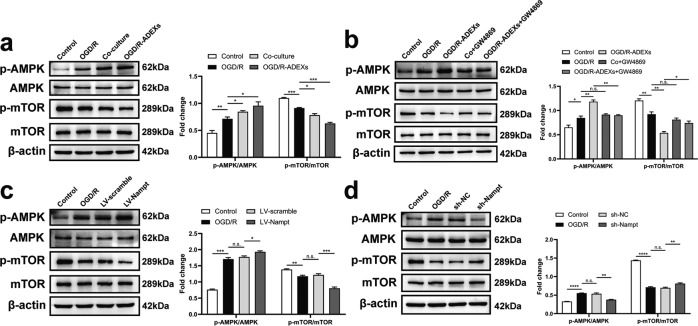
OGD/R-ADEX enhances autophagy through AMPK-mTOR signaling. **a** The expressions of p-AMPK/AMPK and p-mTOR/mTOR were determined by western blotting of OGD/R-induced neurons treated with OGD/R astrocytes or OGD/R-ADEXs (*n* = 5). **b** The expressions of p-AMPK/AMPK and p-mTOR/mTOR were determined by western blotting of OGD/R-induced neurons treated with OGD/R-ADEXs, Co+GW, or OGD/R-ADEXs+GW (*n* = 5). **c** The expressions of p-AMPK/AMPK and p-mTOR/mTOR were determined by western blotting of OGD/R-induced neurons transfected with LV-Nampt or with LV-scramble (*n* = 5). **d** The expressions of p-AMPK/AMPK and p-mTOR/mTOR were determined by western blotting of OGD/R-induced neurons transfected with sh-Nampt or sh-NC (*n* = 5). Data are shown as the mean ± SD of at least three independent experiments. ^*^P < 0.05; ^**^P < 0.01; ^***^P < 0.001; ^****^P < 0.0001. Neurons incubated under standard cell culture conditions (Control) were used as negative control; Neurons treated with PBS under OGD/R conditions served as positive control (OGD/R); OGD/R-ADEXs, exosomes isolated from OGD/R astrocytes; Co-culture, OGD/R neurons co-cultured with OGD/R astrocytes; Co+GW4869, OGD/R astrocytes treated with the exosome secretion inhibitor GW4869; OGD/R-ADEXs+GW4869, OGD/R-ADEXs isolated from OGD/R astrocytes pre‐treated with GW4869. ns, not significant.

To determine how OGD/R-ADEXs regulated autophagy, we characterized signaling cascades associated with autophagy. The AMPK/mTOR signaling pathway is known to regulate autophagy after cerebral ischemia [37]. As expected, neurons incubated with either OGD/R astrocytes or with OGD/R-ADEXs remarkably increased OGD/R-induced upregulation of p-AMPK and downregulation of p-mTOR (Fig. 5a). Importantly, incubation with OGD/R-ADEXs up-regulated the abundance of p-AMPK and downregulated p-mTOR expression in OGD/R-induced neurons, while neurons incubated with exosomes isolated from OGD/R astrocytes pretreated with GW4869 failed to regulate p-AMPK and p-mTOR protein levels (Fig. 5b). In a similar manner, we used LV-Nampt and sh-Nampt directly in OGD/R-induced neurons to analyze the effects of Nampt on the regulation of p-AMPK and p-mTOR. Consistent with autophagy regulation, the protein expression of p-AMPK was increased and p-mTOR was decreased after LV-Nampt transfection (Fig. 5c), whereas levels of these proteins were reversed when the neurons were transfected with sh-Nampt (Fig. 5d). Together, these data indicated that OGD/R-ADEXs possibly modulate neuronal autophagy through AMPK/mTOR signaling in vitro.

### OGD/R-ADEXs promote the recovery of neurological function in mice after strokes

**Fig. 6:**
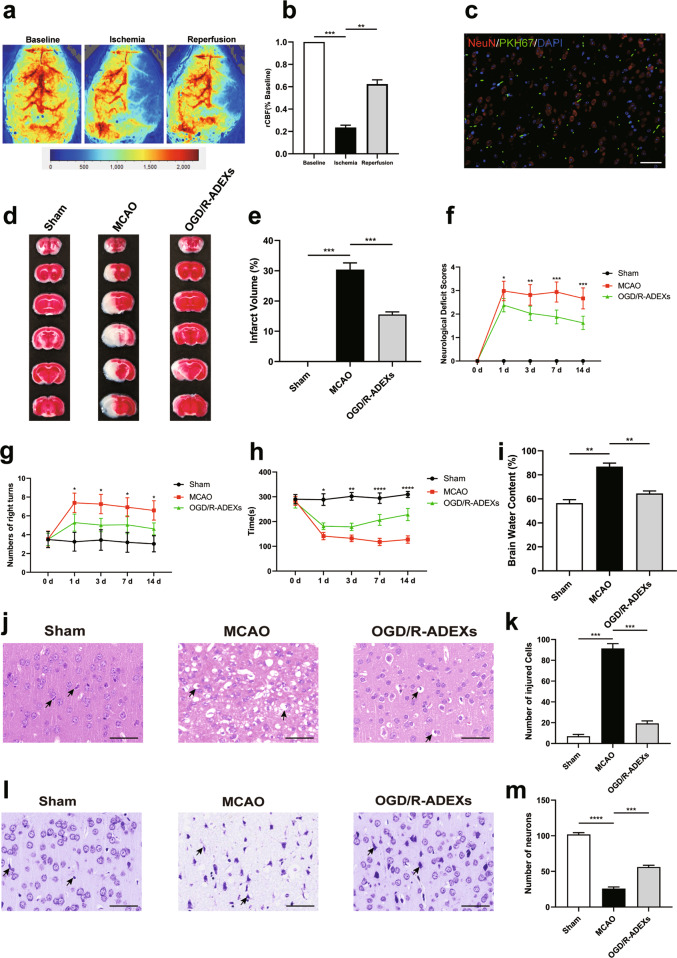
OGD/R-ADEXs reduce brain injury and improve neurological function recovery after stroke. **a**, **b** Representative images of cerebral blood flow (CBF) and the percentage change of rCBF compared with baseline during MCAO/R in occlusion and reperfusion. **c** Representative immunofluorescence images showing the biodistribution of OGD/R-ADEXs within the ischemic cortex. Scale bar: 1 μm. **d** Neuroprotective effects of OGD/R-ADEXs were evaluated by TTC staining (*n* = 5). **e** Quantitation of the infarct volume is shown in (**d**). **f** The neurological deficit score was determined in MCAO/R mice treated with OGD/R-ADEXs (*n* = 5). **g**, **h** The sensorimotor function of mice was evaluated using the Corner (**g**) and rotarod tests (**h**) in MCAO/R mice treated with OGD/R-ADEXs (*n* = 5). **i** The brain water content was measured in MCAO/R mice treated with OGD/R-ADEXs (*n* = 5). **j**, **k** Photomicrograph of mice brain tissue region using hematoxylin and eosin staining of MCAO/R mice brains from mice treated with OGD/R-ADEXs (*n* = 5). Scale bar: 50 μm. **l**, **m** Representative images of Nissl bodies in the ischemic cortex in MCAO/R mice treated with OGD/R-ADEXs (*n* = 5). Data are shown as the mean ± SD of at least three independent experiments. ^*^P < 0.05; ^**^P < 0.01; ^***^P < 0.001; ^****^P < 0.0001. Mice underwent the surgery except that the filament was not inserted into ICA (Sham); Mice was exposed to MCAO followed by administration of PBS (MCAO); OGD/R-ADEXs, exosomes isolated from OGD/R astrocytes.

Based on the above mentioned in vitro results of neurons, we determined whether treatment with OGD/R-ADEXs could improve neurological recovery after stroke by regulating autophagic activity. The middle cerebral artery occlusion (MCAO) model was shown to exhibit reperfusion, with cerebral blood flow (CBF) decreasing to 21.3 ± 2.33%, and subsequently increasing to 63.3 ± 4.89%, when compared with baseline (Fig. 6a, b). We then found that OGD/R-ADEXs reached the central nervous system when the MCAO mouse model was injected with OGD/R-ADEXs via the tail vein (Fig. 6c). Triphenyl tetrazolium chloride (TTC) staining was used to stain brain coronal slices to measure infarct volumes. Mice that immediately received OGD/R-ADEXs at the beginning of reperfusion reduced infarct volumes (Fig. 6d, e). Along with reduction of infarct volumes, the neurological deficit score showed that OGD/R-ADEXs treatment decreased the neurological deficit score (Fig. 6f). In a similar manner, analysis of the behavioral test results showed that mice treated with OGD/R-ADEXs had better test scores at all time points (Fig. 6g, h). We then analyzed the brain water content after stroke. Consistent with the reduction of neurological injury, decreased brain water content was found in mice treated with OGD/R-ADEXs (Fig. 6i). Furthermore, histological changes and neuronal damage of mice were assessed using hematoxylin and eosin (HE) staining and Nissl staining, respectively. Figure 6j–m shows that ischemic stroke augmented brain damage, while treatment with OGD/R-ADEXs reduced the number of injured cells and neuronal loss. These results conclusively showed that OGD/R-ADEXs reduced brain tissue injury and promoted the recovery of neurological function in an experimental stroke model.

### OGD/R-ADEXs administration after MCAO enhances autophagy through AMPK/mTOR signaling

**Fig. 7:**
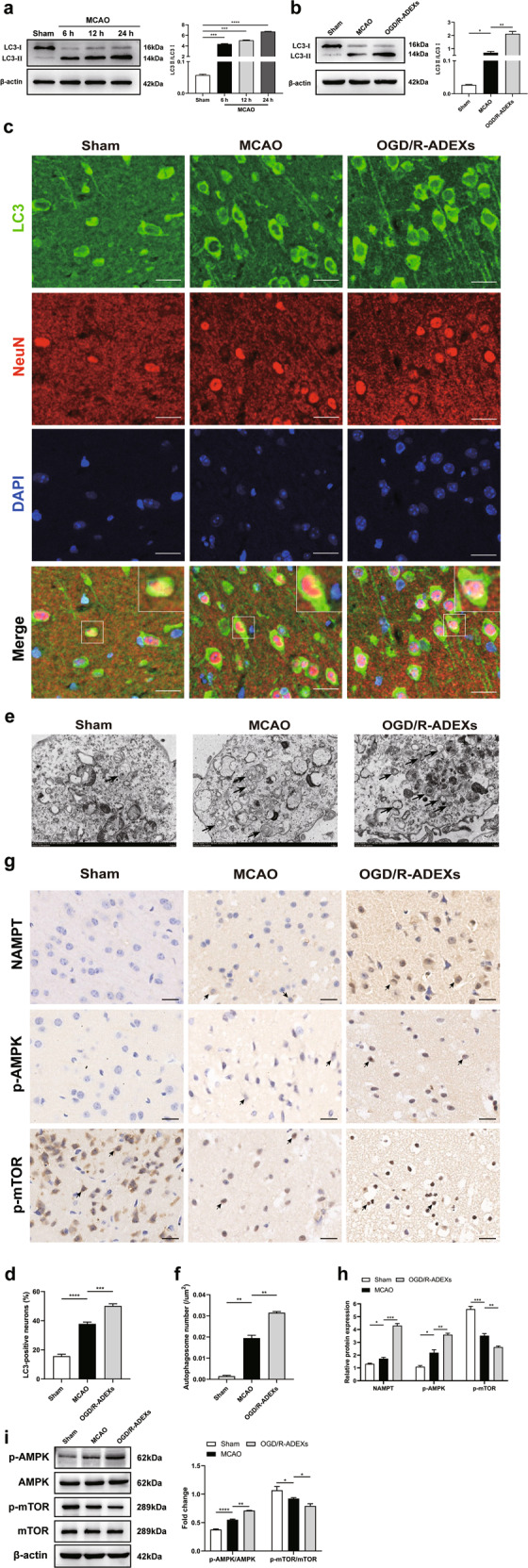
OGD/R-ADEXs injection after MCAO/R enhances autophagy through AMPK/mTOR signaling. **a** The expressions of LC3 II/I were determined by western blotting of sham-operated nice and in MCAO mice after different durations of reperfusions (*n* = 5). **b** The expressions of LC3 II/I were determined by western blotting of MCAO mice injected with OGD/R-ADEXs (*n* = 5). **c**, **d** Representative photomicrographs and quantitation of LC3-positive puncta in MCAO mice injected with OGD/R-ADEXs (*n* = 5). Scale bar: 20 μm. **e**, **f** The autophagosomes were detected by transmission electron microscopy in MCAO mice injected with OGD/R-ADEXs (*n* = 5). Scale bar: 1 μm. **g**, **h** The expressions of Nampt, p-AMPK, and p-mTOR were determined by immunohistochemistry in MCAO mice injected with OGD/R-ADEXs (*n* = 5). Scale bar: 20 μm. **i** The expressions of p-AMPK/AMPK and p-mTOR/mTOR were determined by western blotting of MCAO mice injected with OGD/R-ADEXs (*n* = 5). Data are shown as the mean ± SD of at least three independent experiments. ^*^P < 0.05; ^**^P < 0.01; ^***^P < 0.001; ^****^P < 0.0001. Mice underwent the surgery except that the filament was not inserted into ICA (Sham); Mice was exposed to MCAO followed by administration of PBS (MCAO); OGD/R-ADEXs, exosomes isolated from OGD/R astrocytes.

To further determine whether the neuroprotective effects of OGD/R-ADEXs were related to autophagy regulation in vivo, we measured the autophagy levels in the untreated MCAO model mice at different time periods. Western blotting showed that compared with sham animals, the protein levels of LC3 II/I peaked at 24 h after stroke (Fig. 7a). Mice treated with OGD/R-ADEXs at the beginning of reperfusion showed a significant increase in autophagy expression at 24 h post-stroke (Fig. 7b–f). These results indicated that OGD/R-ADEXs promoted autophagy in a mouse model of stroke. We then detected the expressions of p-AMPK and p-mTOR in vivo. In line with our in vitro results, p-AMPK protein expression was increased and p-mTOR was decreased in the infarct areas of MCAO mice. Injection of OGD/R-ADEXs at the beginning of reperfusion increased protein levels of p-AMPK and decreased p-mTOR levels in the model mice (Fig. 7g–i). Overall, these results suggested that the AMPK/mTOR signaling pathway participated in the effects of OGD/R-ADEXs in promoting neuronal autophagy in vivo.

### The regulation of OGD/R-ADEXS on autophagy after stroke depends on Nampt

**Fig. 8:**
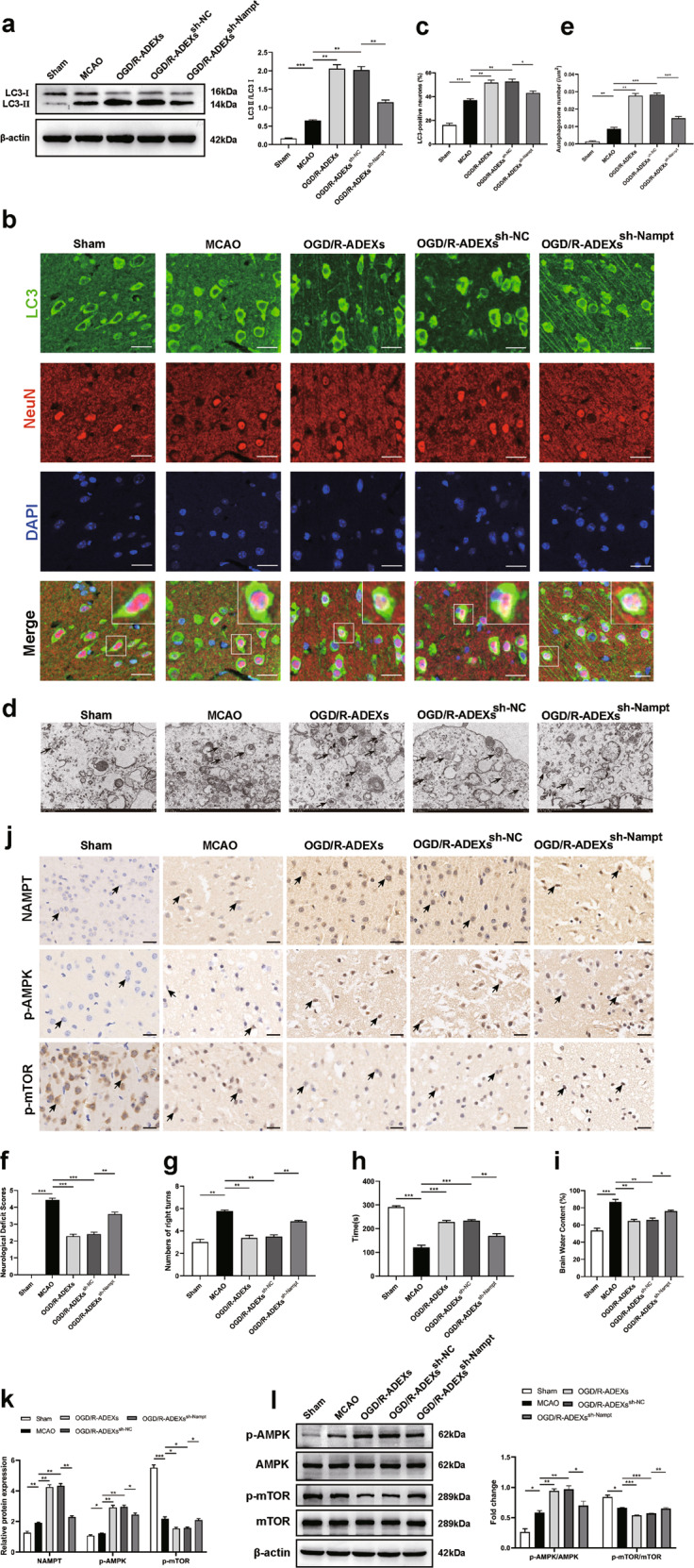
Inhibition of Nampt in OGD/R-ADEXs diminishes post-stroke exosome-induced regulation of autophagy and neuroprotection. **a** The expressions of LC3 II/I were determined using western blotting in MCAO mice injected with OGD/R-ADEXs, OGD/R-ADEXs^sh-Nampt^, or OGD/R-ADEXs^sh-NC^ (*n* = 5). **b**, **c** Representative photomicrographs and quantitation of LC3-positive puncta in MCAO mice injected with OGD/R-ADEXs, OGD/R-ADEXs^sh-Nampt^, or OGD/R-ADEXs^sh-NC^ (*n* = 5). Scale bar: 20 μm. **d**, **e** The autophagosomes were detected by transmission electron microscopy in MCAO mice injected with OGD/R-ADEXs, OGD/R-ADEXs^sh-Nampt^, or OGD/R-ADEXs^sh-NC^ (*n* = 5). Scale bar: 1 μm. **f** The neurological deficit score was measured in MCAO mice treated with OGD/R-ADEXs, OGD/R-ADEXs^sh-Nampt^, or OGD/R-ADEXs^sh-NC^ (*n* = 5). **g**, **h** The sensorimotor function of mice was evaluated using the corner (**g**) and rotarod tests (**h**) in MCAO mice treated with OGD/R-ADEXs, OGD/R-ADEXs^sh-Nampt^, or OGD/R-ADEXs^sh-NC^. **i** The brain water content was measured in mice treated with OGD/R-ADEXs, OGD/R-ADEXs^sh-Nampt^, or OGD/R-ADEXs^sh-NC^ (*n* = 5). **j**, **k** The expressions of Nampt, p-AMPK, or p-mTOR were determined using immunohistochemistry in MCAO mice injected with OGD/R-ADEXs, OGD/R-ADEXs^sh-Nampt^, or OGD/R-ADEXs^sh-NC^ (*n* = 5). Scale bar: 20 μm. **l** The expressions of p-AMPK/AMPK and p-mTOR/mTOR were determined using western blotting of MCAO mice injected with OGD/R-ADEXs, OGD/R-ADEXs^sh-Nampt^, or OGD/R-ADEXs^sh-NC^ (*n* = 5). Data are shown as the mean ± SD of at least three independent experiments. ^*^P < 0.05; ^**^P < 0.01; ^***^P < 0.001. Mice underwent the surgery except that the filament was not inserted into ICA (Sham); Mice was exposed to MCAO followed by administration of PBS (MCAO); OGD/R-ADEXs, exosomes isolated from OGD/R astrocytes. OGD/R-ADEXs^sh-Nampt^, exosomes isolated from OGD/R astrocytes that were pretreated with sh-Nampt; OGD/R-ADEXs^sh-NC^, exosomes isolated from OGD/R astrocytes that were pretreated with scramble.

To verify that the increased autophagy expression induced by OGD/R-ADEXs was caused by exosomal delivery of Nampt, we injected either PBS, OGD/R-ADEXs, or OGD/R-ADEXs pretreated with sh-Nampt (OGD/R-ADEXs^sh-Nampt^), or with OGD/R-ADEXs pretreated with the control (OGD/R-ADEXs^sh-NC^) into the stroke model mice. The OGD/R-ADEXs and OGD/R-ADEXs^sh-NC^ groups showed higher expressions of autophagy in the ischemic cortex, when compared with the PBS group. However, injection of OGD/R-ADEXs^sh-Nampt^ remarkably reduced the autophagy levels of neurons, when compared with OGD/R-ADEXs^sh-NC^ (Fig. 8a–e). The neurological deficit scores of mice treated with OGD/R-ADEXs^sh-Nampt^ consistently increased, when compared with that of the OGD/R-ADEXs^sh-NC^ treatment group (Fig. 8f). The corner and rotarod tests indicated that treatment with OGD/R-ADEXs^sh-NC^ enhanced neurological recovery in the model mice, which was specifically reversed by OGD/R-ADEXs^sh-Nampt^ (Fig. 8g–h). In a similar manner, the brain water content was higher in the OGD/R-ADEXs^sh-Nampt^ group, when compared with the OGD/R-ADEXs^sh-NC^ group (Fig. 8i). Immunohistochemistry and western blot analyses showed that mice treated with OGD/R-ADEXs^sh-Nampt^ showed decreased protein abundance of p-AMPK and increased protein abundance of p-mTOR, when compared with animals treated with OGD/R-ADEXs^sh-NC^ (Fig. 8j–l). These results conclusively showed that the promoting effect of OGD/R-ADEXs on autophagy in model mice was at least partially mediated by Nampt transferred by exosomes.

## Discussion

**Fig. 9:**
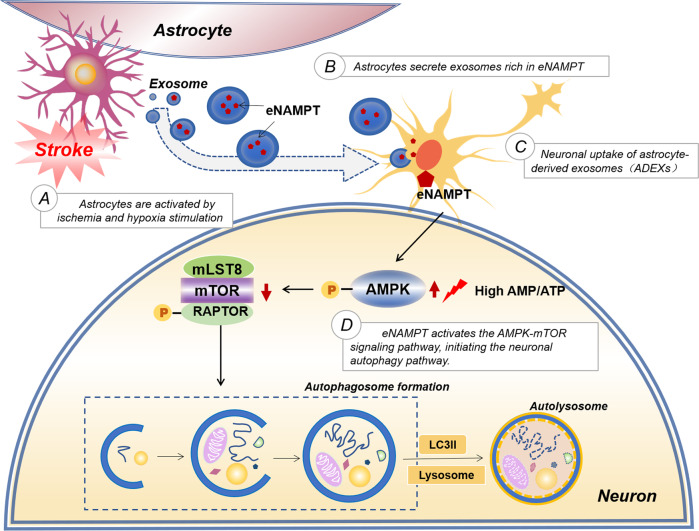
A schematic diagram showing the neuroprotective effects of Nampt transferred by OGD/R-ADEXs in an experimental stroke model. After OGD/R induction, astrocytes release exosomes enriched with Nampt, which are internalized by neurons. In neurons, Nampt leads to the upregulation of the p-AMPK protein levels, followed by a reduction of p-mTOR levels. The inhibition of p-mTOR subsequently further enhances the levels of neuronal autophagy.

Exosomes from various cells have neuroprotective effects in experimental stroke models. However, the underlying mechanisms of beneficial effects induced by exosomes during stroke conditions are still poorly defined. The present study used in vitro and in vivo stroke models to show that exosomes derived from OGD/R astrocytes alleviated neuronal injury and promoted neurological recovery. Further results indicated that this effect was closely related to increasing neuronal autophagy levels after ischemia. A crucial mechanism for OGD/R astrocytes in promoting neuroprotection involved transfer of Nampt from astrocytes to neurons via exosomes, thereby activating the AMPK/mTOR signaling pathway (Fig. 9).

Many signaling pathways have been related to the occurrence and development of cerebral ischemic disease [38]. Recent studies reported that, among these different mechanisms, autophagy may be a key target for stroke treatment [39, 40]. Our results keep in accordance with previous results, showing that neuronal autophagy was rapidly activated during either MCAO/reperfusion (MCAO/R) or oxygen-glucose deprivation/reoxygenation (OGD/R). In addition, autophagy after stroke was a dynamic process, reaching its peak at 24 h after MCAO treatment. According to previous reports and our present study, autophagy is vital to neuronal function in both physiological and pathological conditions, with autophagy levels moderately increasing under mild stimulation conditions such as ischemia, which showed that neurons can still maintain sufficient energy [41]. Nevertheless, exacerbation of the disease process led to overactivation of autophagy, which ultimately resulted in unprogrammed cell death [42, 43]. These studies indicate that the well-balanced regulation of autophagy after stroke is very important [27].

Exosomes are extracellular vesicles with a diameter of 50–140 nm formed by fusion of the plasma membrane [44]. As an important intercellular communication medium, exosomes have been reported to be released by the central nervous system cells, and have the potential to treat ischemic stroke [45–48], but this possibility has only recently been elucidated. Previous studies have suggested that exosomes have pro-angiogenic, anti-inflammatory, and anti-apoptosis properties [49–52]. As previously reported, exosomes can transport miRNAs [53], as well as diverse sets of proteins [54–56]. Intercellular transfer of proteins by exosomes is a relatively new research field involving proteins present in tiny cellular particles in diverse disease systems, which will be of great significance. Nampt is an important rate-limiting enzyme in the synthesis of nicotinamide adenine dinucleotide from nicotinamide in mammals. It has traditionally been considered to play a vital role in obesity, inflammation, aging, diabetes, and motor dysfunction. It has also been shown that Nampt is up-regulated in neurons induced by the oxygen-glucose deprivation (OGD) model [18] in vitro, as well as in animals with the MCAO model in vivo [57, 58]. A recent study by Lu et al. [36] determined that microglia actively secreted Nampt through exosomes during inflammation after ischemic stroke. We therefore hypothesized that Nampt-expressing astrocytes also transferred Nampt via this pathway, thereby protecting neurons and brain tissue. The results of the present study proved that OGD/R-ADEXs decreased cerebral ischemia injury and improved neurological function by promoting neuronal autophagy, and that the key compound exerting this effect was Nampt. To verify that Nampt was a key enzyme in mediating the neuroprotective effects of OGD/R-ADEXs, we injected OGD/R-ADEXs pretreated with sh-Nampt into stroke model mice via the tail vein. As expected, knockdown of Nampt eliminated the protective effects of OGD/R-ADEXs, as demonstrated by the reduction of neuronal damage and improvement of behavioral tests.

It is well-known that the key to successful autophagy regulation involves an accurate autophagy signaling pathway that is inhibited or promoted. Several studies have indicated that AMPK/mTOR signaling was related with autophagy [59], and played a critical role in ischemic stroke [60]. Phosphorylated adenosine monophosphate-activated protein kinase (p-AMPK) could inhibit the activation of the mammalian target of rapamycin (mTOR) [61]. In this present study, p-AMPK protein levels were increased and p-mTOR protein levels were decreased after acute cerebral ischemia, and OGD/R-ADEX reversed the expression levels of these proteins. Overall, the results of our study indicated that the AMPK/mTOR signaling pathway played an important role in neuroprotection produced by OGD/R-ADEXs in the early stage of ischemic stroke, but we could not completely rule out whether OGD/R-ADEXs regulated other autophagy-related signaling pathways.

Notably, there are some limitations in this study. Firstly, although our data indicated that Nampt in the OGD/R-ADEXs was significantly increased and indirectly proved that Nampt was located inside the exosomes by western blot, we did not provide direct evidence for intra-exosomal localization of Nampt. Immunogold labeling should be used to visualize the localization of Nampt within exosomes in the future. Secondly, in our present study, we did not explore in depth whether the increase of autophagy is due to the upregulation of autophagosome formation or blockage of autophagic degradation. Thus, Lysosome protease inhibition test should be performed to distinguish these in the future. Thirdly, we only focused on exploring the effects of OGD/R-ADEXs on neurons under brain ischemia conditions. In our future studies, it is necessary to compare the difference in the roles of ADEXs under physiological conditions and OGD/R-ADEXs under pathological conditions in ischemic stroke.

To summarize, our study provided new insight into the protective effects and mechanisms of exosomes of astrocytes during acute ischemic stroke. The results showed that OGD/R-ADEXs produced neuroprotective effects and promoted neurofunctional recovery by enhancing ischemia-induced neuronal autophagy. The increase of neuronal autophagy resulted from astrocytes transporting Nampt to neurons via exosomes, followed by Nampt-regulated neuronal autophagy using the AMPK/mTOR signaling pathway. These novel findings on exosomes secreted by OGD/R-induced astrocytes could provide new strategies for the development of novel therapeutic targets to treat AIS.

## Supplementary information

Sequences used for knockdown of target genes

Experimental groups and survival rates of mice

Supplemental figure legend

Supplementary Fig. 1

Supplementary Fig. 2

DECLARATION OF CONTRIBUTIONS TO ARTICLE

Reproducibility Checklist

## Data Availability

The datasets used or analyzed during the study are available from the corresponding author on reasonable request. All data generated or analyzed during this study are included in this published article and its supplementary information files.

## References

[1] Phipps MS, Cronin CA (2020). Management of acute ischemic stroke. BMJ.

[2] Blakeley JO, Llinas RH (2007). Thrombolytic therapy for acute ischemic stroke. J Neurol Sci.

[3] Prabhakaran S, Ruff I, Bernstein RA (2015). Acute stroke intervention: a systematic review. JAMA.

[4] Ma YL, Zhang LX, Liu GL, Fan Y, Peng Y, Hou WG (2017). N-Myc downstream-regulated gene 2 (Ndrg2) is involved in ischemia-hypoxia-induced astrocyte apoptosis: a novel target for stroke therapy. Mol Neurobiol.

[5] Yamagata K (2021). Astrocyte-induced synapse formation and ischemic stroke. J Neurosci Res.

[6] Jiang MQ, Yu SP, Wei ZZ, Zhong W, Cao W, Gu X (2021). Conversion of reactive astrocytes to induced neurons enhances neuronal repair and functional recovery after ischemic stroke. Front Aging Neurosci.

[7] Théry C, Zitvogel L, Amigorena S (2002). Exosomes: composition, biogenesis and function. Nat Rev Immunol.

[8] Deng Z, Wang J, Xiao Y, Li F, Niu L, Liu X (2021). Ultrasound-mediated augmented exosome release from astrocytes alleviates amyloid-β-induced neurotoxicity. Theranostics.

[9] Du L, Jiang Y, Sun Y (2021). Astrocyte-derived exosomes carry microRNA-17-5p to protect neonatal rats from hypoxic-ischemic brain damage via inhibiting BNIP-2 expression. Neurotoxicology.

[10] Long X, Yao X, Jiang Q, Yang Y, He X, Tian W (2020). Astrocyte-derived exosomes enriched with miR-873a-5p inhibit neuroinflammation via microglia phenotype modulation after traumatic brain injury. J Neuroinflammation.

[11] Shakespear N, Ogura M, Yamaki J, Homma Y (2020). Astrocyte-derived exosomal microRNA miR-200a-3p prevents MPP-induced apoptotic cell death through down-regulation of MKK4. Neurochem Res.

[12] Chen Y, Xia K, Chen L, Fan D (2019). Increased interleukin-6 Levels in the astrocyte-derived exosomes of sporadic amyotrophic lateral sclerosis patients. Front Neurosci.

[13] Adolf A, Rohrbeck A, MünsterWandowski A, Johansson M, Kuhn H-G, Kopp MA (2019). Release of astroglial vimentin by extracellular vesicles: Modulation of binding and internalization of C3 transferase in astrocytes and neurons. Glia.

[14] Hong Y, Zhao T, Li XJ, Li S (2017). Mutant huntingtin inhibits αB-crystallin expression and impairs exosome secretion from astrocytes. J Neurosci.

[15] Pei X, Li Y, Zhu L, Zhou Z (2020). Astrocyte-derived exosomes transfer miR-190b to inhibit oxygen and glucose deprivation-induced autophagy and neuronal apoptosis. Cell Cycle.

[16] Wu W, Liu J, Yang C, Xu Z, Huang J, Lin J (2020). Astrocyte-derived exosome-transported microRNA-34c is neuroprotective against cerebral ischemia/reperfusion injury via TLR7 and the NF-κB/MAPK pathways. Brain Res Bull.

[17] Wang SN, Miao CY (2019). Targeting NAMPT as a therapeutic strategy against stroke. Stroke Vasc Neurol.

[18] Wang P, Xu TY, Guan YF, Tian W-W, Viollet B, Rui YC (2011). Nicotinamide phosphoribosyltransferase protects against ischemic stroke through SIRT1-dependent adenosine monophosphate-activated kinase pathway. Ann Neurol.

[19] Wang P, Shao BZ, Deng Z, Chen S, Yue Z, Miao CY (2018). Autophagy in ischemic stroke. Prog Neurobiol.

[20] Chen W, Sun Y, Liu K, Sun X (2014). Autophagy: a double-edged sword for neuronal survival after cerebral ischemia. Neural Regen Res.

[21] Carloni S, Girelli S, Scopa C, Buonocore G, Longini M, Balduini W (2010). Activation of autophagy and Akt/CREB signaling play an equivalent role in the neuroprotective effect of rapamycin in neonatal hypoxia-ischemia. Autophagy.

[22] Dai SH, Chen T, Li X, Yue KY, Luo P, Yang LK (2017). Sirt3 confers protection against neuronal ischemia by inducing autophagy: involvement of the AMPK-mTOR pathway. Free Radic Biol Med.

[23] Song DD, Zhang TT, Chen JL, Xia YF, Qin ZH, Waeber C (2017). Sphingosine kinase 2 activates autophagy and protects neurons against ischemic injury through interaction with Bcl-2 via its putative BH3 domain. Cell Death Dis.

[24] Yan BC, Wang J, Rui Y, Cao J, Xu P, Jiang D (2019). Neuroprotective effects of gabapentin against cerebral ischemia reperfusion-induced neuronal autophagic injury via regulation of the PI3K/Akt/mTOR signaling pathways. J Neuropathol Exp Neurol.

[25] He S, Wang C, Dong H, Xia F, Zhou H, Jiang X (2012). Immune-related GTPase M (IRGM1) regulates neuronal autophagy in a mouse model of stroke. Autophagy.

[26] Mo ZT, Fang YQ, He YP, Zhang S (2012). β-Asarone protects PC12 cells against OGD/R-induced injury via attenuating Beclin-1-dependent autophagy. Acta Pharm Sin.

[27] Shi R, Weng J, Zhao L, Li XM, Gao TM, Kong J (2012). Excessive autophagy contributes to neuron death in cerebral ischemia. CNS Neurosci Ther.

[28] Xin XY, Pan J, Wang XQ, Ma JF, Ding JQ, Yang GY (2011). 2-methoxyestradiol attenuates autophagy activation after global ischemia. Can J Neurol Sci.

[29] Thompson CA, Purushothaman A, Ramani VC, Vlodavsky I, Sanderson RD (2013). Heparanase regulates secretion, composition, and function of tumor cell-derived exosomes. J Biol Chem.

[30] Santangelo L, Giurato G, Cicchini C, Montaldo C, Mancone C, Tarallo R (2016). The RNA-binding protein SYNCRIP is a component of the hepatocyte exosomal machinery controlling microrna sorting. Cell Rep.

[31] Zheng Y, Zhao P, Lian Y, Li S, Chen Y, Li L (2020). MiR-340-5p alleviates oxygen-glucose deprivation/reoxygenation-induced neuronal injury via PI3K/Akt activation by targeting PDCD4. Neurochem Int.

[32] Longa EZ, Weinstein PR, Carlson S, Cummins R (1989). Reversible middle cerebral artery occlusion without craniectomy in rats. Stroke.

[33] Yang Z, Weian C, Susu H, Hanmin W (2016). Protective effects of mangiferin on cerebral ischemia-reperfusion injury and its mechanisms. Eur J Pharm.

[34] Zheng X, Zhang L, Kuang Y, Venkataramani V, Jin F, Hein K (2021). Extracellular vesicles derived from neural progenitor cells–a preclinical evaluation for stroke treatment in mice. Transl Stroke Res.

[35] Tamai K, Tanaka N, Nakano T, Kakazu E, Kondo Y, Inoue J (2010). Exosome secretion of dendritic cells is regulated by Hrs, an ESCRT-0 protein. Biochem Biophys Res Commun.

[36] Lu YB, Chen CX, Huang J, Tian YX, Xie X, Yang P (2019). Nicotinamide phosphoribosyltransferase secreted from microglia via exosome during ischemic injury. J Neurochem.

[37] Hwang JY, Gertner M, Pontarelli F, CourtVazquez B, Bennett MVL, Ofengeim D (2017). Global ischemia induces lysosomal-mediated degradation of mTOR and activation of autophagy in hippocampal neurons destined to die. Cell death Differ.

[38] Fricker M, Tolkovsky AM, Borutaite V, Coleman M, Brown GC (2018). Neuronal cell death. Physiol Rev.

[39] Descloux C, Ginet V, Clarke PGH, Puyal J, Truttmann AC (2015). Neuronal death after perinatal cerebral hypoxia-ischemia: focus on autophagy-mediated cell death. Int J Dev Neurosci.

[40] Puyal J, Ginet V, Grishchuk Y, Truttmann AC, Clarke PGH (2012). Neuronal autophagy as a mediator of life and death: contrasting roles in chronic neurodegenerative and acute neural disorders. Neuroscientist.

[41] Kim KA, Shin D, Kim JH, Shin YJ, Rajanikant GK, Majid A (2018). Role of autophagy in endothelial damage and blood-brain barrier disruption in ischemic Stroke. Stroke.

[42] Ginet V, Spiehlmann A, Rummel C, Rudinskiy N, Grishchuk Y, LuthiCarter R (2014). Involvement of autophagy in hypoxic-excitotoxic neuronal death. Autophagy.

[43] Liu Y, Levine B (2015). Autosis and autophagic cell death: the dark side of autophagy. Cell death Differ.

[44] Raposo G, Stoorvogel W (2013). Extracellular vesicles: exosomes, microvesicles, and friends. J Cell Biol.

[45] Kang X, Zuo Z, Hong W, Tang H, Geng W (2019). Progress of research on exosomes in the protection against ischemic brain injury. Front Neurosci.

[46] Li Y, Tang Y, Yang GY. Therapeutic application of exosomes in ischaemic stroke. Stroke Vasc Neurol. 2021;6:483–95. 10.1136/svn-2020-000419 PMC8485240 33431513

[47] Ueno Y, Hira K, Miyamoto N, Kijima C, Inaba T, Hattori N. Pleiotropic effects of exosomes as a therapy for stroke recovery. Int J Mol Sci. 2020; 21:6894. 10.3390/ijms21186894 PMC7555640 32962207

[48] Wang W, Li Z, Feng J (2018). The potential role of exosomes in the diagnosis and therapy of ischemic diseases. Cytotherapy.

[49] Geng W, Tang H, Luo S, Lv Y, Liang D, Kang X (2019). Exosomes from miRNA-126-modified ADSCs promotes functional recovery after stroke in rats by improving neurogenesis and suppressing microglia activation. Am J Transl Res.

[50] Duan S, Wang F, Cao J, Wang C (2020). Exosomes derived from microRNA-146a-5p-enriched bone marrow mesenchymal stem cells alleviate intracerebral hemorrhage by inhibiting neuronal apoptosis and microglial M1 polarization. Drug Des Devel Ther.

[51] Zhang H, Wu J, Wu J, Fan Q, Zhou J, Wu J (2019). Exosome-mediated targeted delivery of miR-210 for angiogenic therapy after cerebral ischemia in mice. J Nanobiotechnology.

[52] Xiao Y, Geng F, Wang G, Li X, Zhu J, Zhu W. Bone marrow-derived mesenchymal stem cells-derived exosomes prevent oligodendrocyte apoptosis through exosomal miR-134 by targeting caspase-8. J Cell Biochem. 2018 10.1002/jcb.27519. 10.1002/jcb.27519 30191592

[53] Ghoreishy A, Khosravi A, Ghaemmaghami A (2019). Exosomal microRNA and stroke: a review. J Cell Biochem.

[54] Li X, Zhang Y, Wang Y, Zhao D, Sun C, Zhou S (2020). Exosomes derived from CXCR4-overexpressing BMSC promoted activation of microvascular endothelial cells in cerebral ischemia/reperfusion injury. Neural Plast.

[55] Shi C, UlkeLemée A, Deng J, Batulan Z, O’brien ER (2019). Characterization of heat shock protein 27 in extracellular vesicles: a potential anti-inflammatory therapy. FASEB J.

[56] Goetzl EJ, Schwartz JB, Mustapic M, Lobach IV, Daneman R, Abner EL (2017). Altered cargo proteins of human plasma endothelial cell-derived exosomes in atherosclerotic cerebrovascular disease. FASEB J.

[57] Jing Z, Xing J, Chen X, Stetler RA, Weng Z, Gan Y (2014). Neuronal NAMPT is released after cerebral ischemia and protects against white matter injury. J Cereb Blood Flow Metab.

[58] Zhao Y, Liu XZ, Tian WW, Guan YF, Wang P, Miao CY (2014). Extracellular visfatin has nicotinamide phosphoribosyltransferase enzymatic activity and is neuroprotective against ischemic injury. CNS Neurosci Ther.

[59] Ha J, Guan KL, Kim J (2015). AMPK and autophagy in glucose/glycogen metabolism. Mol Asp Med.

[60] Jiang S, Li T, Ji T, Yi W, Yang Z, Wang S (2018). AMPK: potential therapeutic target for ischemic stroke. Theranostics.

[61] Leech T, Chattipakorn N, Chattipakorn SC (2019). The beneficial roles of metformin on the brain with cerebral ischaemia/reperfusion injury. Pharm Res.

