# Supplementary material for: Astrocyte-derived exosomal nicotinamide phosphoribosyltransferase (Nampt) ameliorates ischemic stroke injury by targeting AMPK/mTOR signaling to induce autophagy
Source: Cell Death Dis. 2022 Dec 20;13(12):1057. doi: 10.1038/s41419-022-05454-9 (PMC9767935; doi:10.1038/s41419-022-05454-9)
Supplement: Supplementary file 1 — Sequences used for knockdown of target genes [file 41419_2022_5454_MOESM1_ESM.docx]

**Suppl. Table S1. Sequences used for knockdown of target genes**

| **Names** | **sense/anti-sense** | **Sequences 5'-3'** |
| --- | --- | --- |
| si-NC | sense | UCACAACCUCCUAGAAAGAGUAGA |
|  | anti-sense | UACUCUUUCUAGGAGGUUGUUAUU |
| si-Hrs | sense | GGAACUACUGGGAGAAGAAdTdT |
|  | anti-sense | UUCUUCUCCCAGUAGUUCCdGdG |
| sh-NC | sense | TTCTCCGAACGTGTCACGT |
| sh-Nampt | sense | GCATCTGCTCATTTGGTTAAC |
