# Supplementary material for: Astrocyte-derived exosomal nicotinamide phosphoribosyltransferase (Nampt) ameliorates ischemic stroke injury by targeting AMPK/mTOR signaling to induce autophagy
Source: Cell Death Dis. 2022 Dec 20;13(12):1057. doi: 10.1038/s41419-022-05454-9 (PMC9767935; doi:10.1038/s41419-022-05454-9)
Supplement: Supplementary file 2 — Experimental groups and survival rates of mice [file 41419_2022_5454_MOESM2_ESM.docx]

**Suppl. Table S2. Experimental groups and survival rates of mice.**

|  | **BT/BW/IHC/IF/TEM** | | | | **Western blot analysis** | | | | **TTC staining** | | | |
| --- | --- | --- | --- | --- | --- | --- | --- | --- | --- | --- | --- | --- |
| Groups | Number | Dead | Survival | Duration | Number | Dead | Survival | Duration | Number | Dead | Survival | Duration |
| Sham | BT 6  BW 6  IHC/IF 6  TEM 6 | 0  0  0  0 | 100 %  100 %  100 %  100 % | 14 days  1 day  1 day  1 day | 6 | 0 | 100 % | 1 day | 6 | 0 | 100 % | 1 day |
| MCAO | BT 6  BW 6  IHC/IF 6  TEM 6 | 1  1  0  0 | 83.3 %  83.3 %  100 %  100 % | 14 days  1 day  1 day  1 day |  |  |  |  | 6 | 0 | 100 % | 1 day |
| OGD/R-ADEXs | BT 6  BW 6  IHC/IF 6  TEM 6 | 1  0  1  0 | 83.3 %  100 %  83.3 %  100 % | 14 days  1 day  1 day  1 day | 6 | 0 | 100 % | 1 day | 6 | 0 | 100 % | 1 day |
| OGD/R-ADEXs^sh-NC^ | BT 6  BW 6  IHC/IF 6  TEM 6 | 0  1  0  1 | 100 %  83.3 %  100 %  83.3 % | 1 days  1 day  1 day  1 day | 6 | 0 | 100 % | 1 day |  |  |  |  |
| OGD/R-ADEXs^sh-Nampt^ | BT 6  BW 6  IHC/IF 6  TEM 6 | 1  1  0  1 | 83.3 %  83.3 %  100 %  83.3 % | 1 days  1 day  1 day  1 day | 6 | 0 | 100 % | 1 day |  |  |  |  |
| Reperfusion 6 h |  |  |  |  | 6 | 0 | 100 % | 6 h |  |  |  |  |
| Reperfusion 12 h |  |  |  |  | 6 | 0 | 100 % | 12 h |  |  |  |  |
| Reperfusion 24 h |  |  |  |  | 6 | 0 | 100 % | 24 h |  |  |  |  |

A total of 180 mice was used in the in vivo experiments. Two mice in the MCAO group was excluded because of unsuccessful surgery. Two mice in OGD/R-ADEXs group died because of severe strokes. Two mice in the OGD/R-ADEXs^sh-NC^ group and three mice in the OGD/R-ADEXs^sh-Nampt^ group were sacrificed because of severe pain on the second day after surgery. There was no animal dead in the remaining groups before reaching the end time points in question. Abbreviations: BT, Behavior tests; BW, Brain water; IHC, Immunohistochemistry; IF, Immunofluorescence; TEM, Transmission electron microscopy; TTC, Triphenyl tetrazolium chloride; MCAO, middle cerebral artery occlusion; OGD/R-ADEXs, exosomes isolated from OGD/R astrocytes; OGD/R-ADEXs^sh-NC^, exosomes isolated from OGD/R astrocytes that were pretreated with scramble; OGD/R-ADEXs^sh-Nampt^, exosomes isolated from OGD/R astrocytes that were pretreated with sh-Nampt.­­
