# Supplementary material for: Astrocyte-derived exosomal nicotinamide phosphoribosyltransferase (Nampt) ameliorates ischemic stroke injury by targeting AMPK/mTOR signaling to induce autophagy
Source: Cell Death Dis. 2022 Dec 20;13(12):1057. doi: 10.1038/s41419-022-05454-9 (PMC9767935; doi:10.1038/s41419-022-05454-9)
Supplement: Supplementary file 3 — Supplemental figure legend [file 41419_2022_5454_MOESM3_ESM.docx]

**Supplemental figure legend**

**Figure S1. Exosomes are key compounds in promoting autophagy and mediating neuroprotection.**  **a** Exosome uptake was verified using images of PKH67 fluorescence in primary neurons. Scale bar, 10 μm. **b** The expressions of LC3 II/I were determined using western blotting in OGD/R-induced neurons treated with three different doses of OGD/R-ADEXs (n = 5). **c** The neuroprotective effects of three different doses of OGD/R-ADEXs in OGD/R-induced neurons were evaluated using the CCK-8 assay (n = 5). **d** The neuroprotective effects of three different doses of OGD/R-ADEXs in OGD/R-induced neurons were evaluated using the LDH assay (n = 5). **e** Cell viability was detected in OGD/R-induced neurons treated with OGD/R-ADEXs, or OGD/R-ADEXs pretreated with Protease K (+Protease), Triton X-100 (+Triton X), or both of them (+Protease +Triton X) using the CCK-8 assay (n = 5). Data are shown as the mean ± SD of at least three independent experiments. ^*^*P* < 0.05; ^**^*P* < 0.01; ^***^*P* < 0.001; ^****^*P* < 0.0001. ns, not significant.

**Figure S2.** **Nampt reduces autophagy-related cell death in neurons exposed to OGD/R. a** The expressions of LC3 II/I were determined using western blotting of OGD/R-induced neurons treated with LV-Nampt or LV-scramble (n = 5). **b** The cell viability was detected in OGD/R-induced neurons treated with LV-Nampt or LV-scramble using the CCK-8 assay (n = 5). **c** The expressions of LC3 II/I were determined using western blotting of OGD/R-induced neurons treated with sh-Nampt or sh-NC (n = 5). **d** The cell viability was detected in OGD/R-induced neurons treated with sh-Nampt or sh-NC using the CCK-8 assay (n = 5). Data are shown as the mean ± SD of at least three independent experiments. ^*^*P* < 0.05; ^**^*P* < 0.01; ^***^*P* < 0.001. ns, not significant.
