# Supplementary figures and images for: Astrocyte-derived exosomal nicotinamide phosphoribosyltransferase (Nampt) ameliorates ischemic stroke injury by targeting AMPK/mTOR signaling to induce autophagy
Source: Cell Death Dis. 2022 Dec 20;13(12):1057. doi: 10.1038/s41419-022-05454-9 (PMC9767935; doi:10.1038/s41419-022-05454-9)

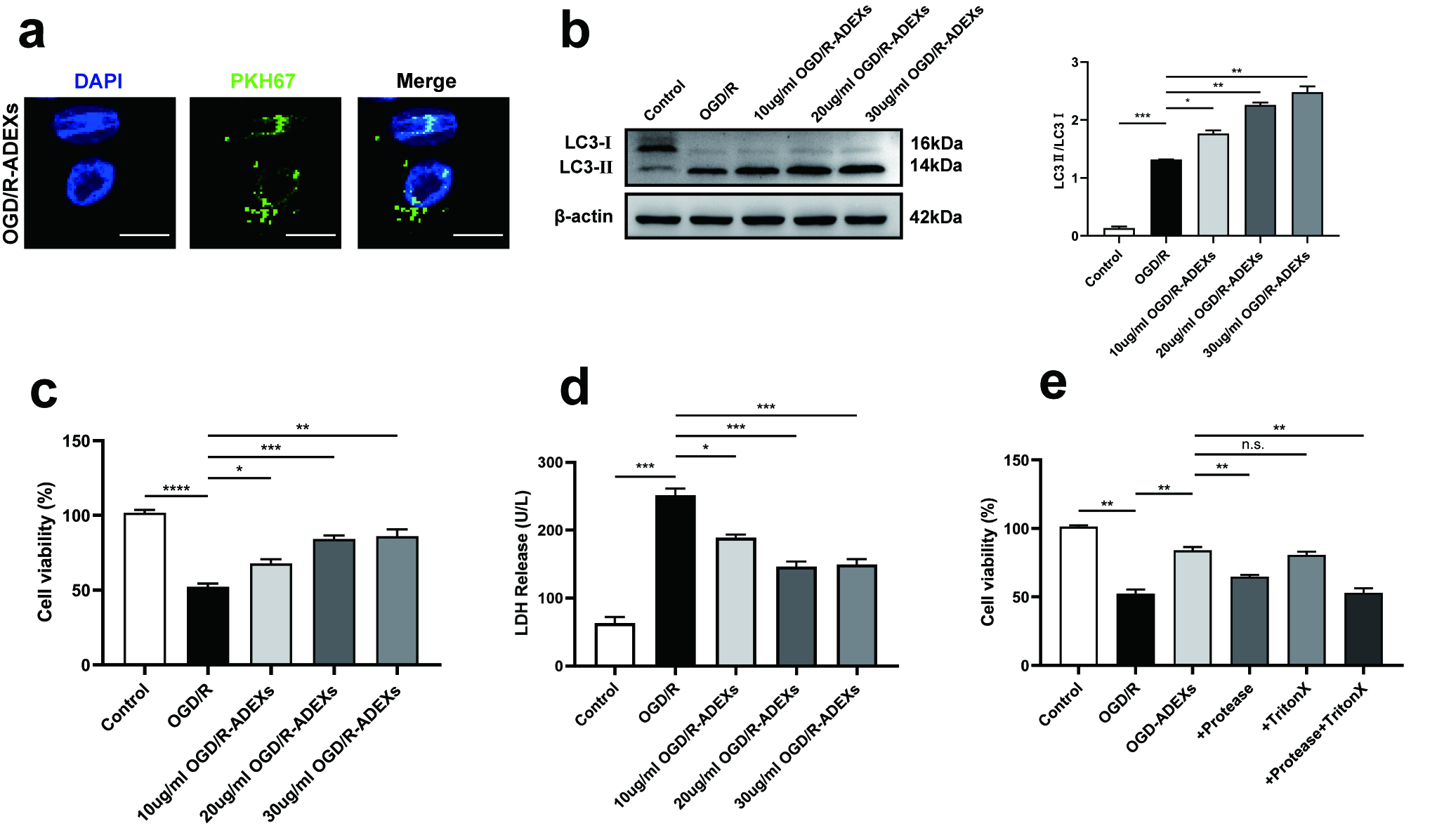

Supplement: Supplementary file 4 — Supplementary Fig. 1 [file 41419_2022_5454_MOESM4_ESM.tif]

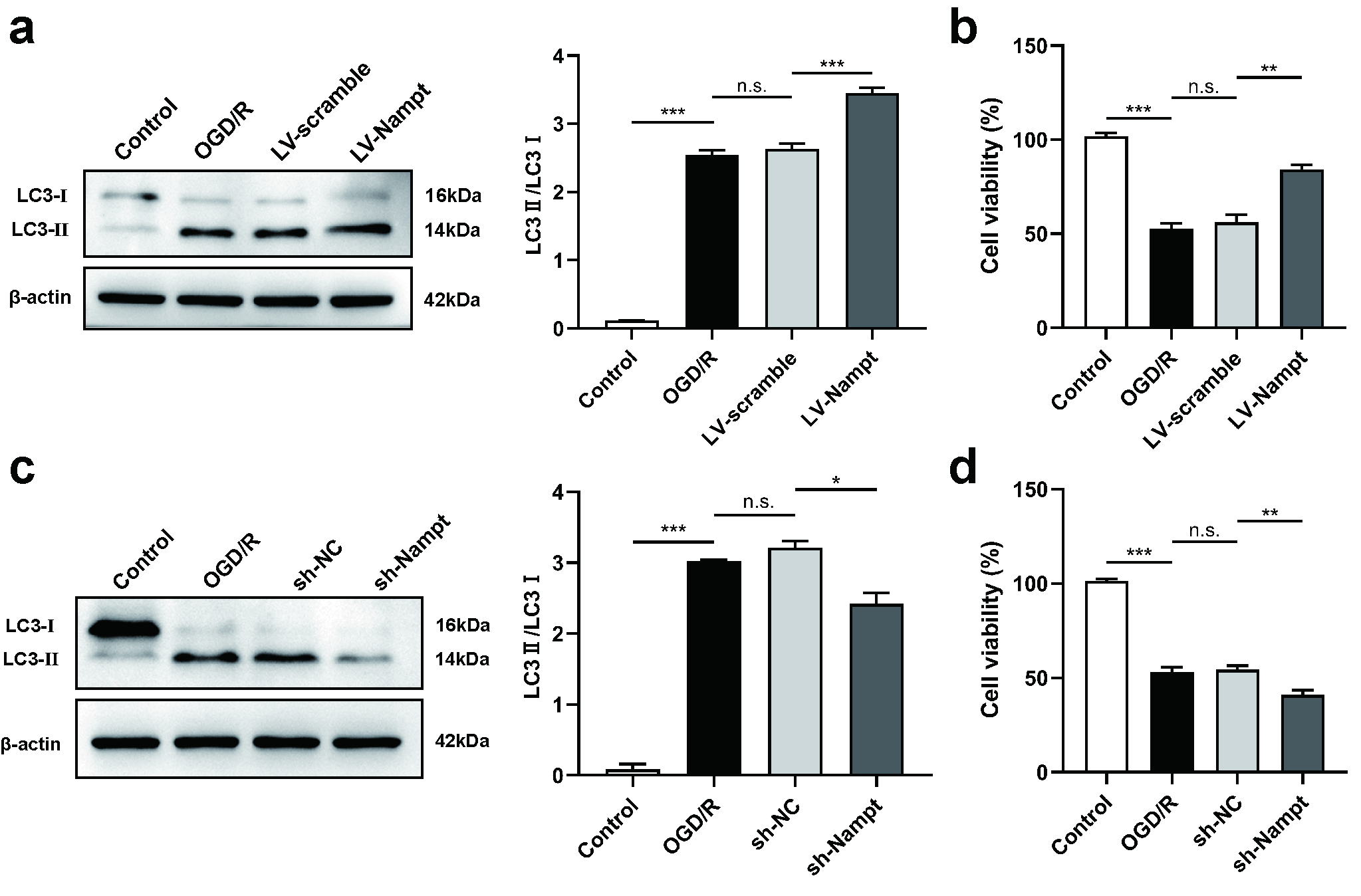

Supplement: Supplementary file 5 — Supplementary Fig. 2 [file 41419_2022_5454_MOESM5_ESM.tif]
