# Supplementary material for: Astrocyte-derived exosomal nicotinamide phosphoribosyltransferase (Nampt) ameliorates ischemic stroke injury by targeting AMPK/mTOR signaling to induce autophagy
Source: Cell Death Dis. 2022 Dec 20;13(12):1057. doi: 10.1038/s41419-022-05454-9 (PMC9767935; doi:10.1038/s41419-022-05454-9)
Supplement: Supplementary file 6 — DECLARATION OF CONTRIBUTIONS TO ARTICLE [file 41419_2022_5454_MOESM6_ESM.pdf]

DECLARATION OF CONTRIBUTIONS TO ARTICLE

ADMC

Manuscript Number:	Journal Name:	
CDDIS-21-3601	*Cell Death & Disease*	(the 'Journal')

Proposed Title of the Contribution:

Astrocyte-derived exosomal nicotinamide phosphoribosyltransferase (Nampt) ameliorates ischemic stroke injury by targeting AMPK/mTOR signaling to induce autophagy (the 'Contribution)

Author(s):

Yang Deng1,2#, Rui Duan3#, Wangli Ding1,2#,Qiuchen Gu1,2 Manman Liu1,2, Junshan Zhou3, Jianguo Sun4*, Junrong Zhu1,2* (the 'Authors')

For all *CDDis* articles, each person named as an author in the published version must be able to show he or she has contributed substantially to the article.

Authorship credit should be based on 1) substantial contributions to conception and design, acquisition of data, or analysis and interpretation of data; 2) drafting the article or revising it critically for important intellectual content; and 3) final approval of the version to be published. Authors should meet conditions 1, 2 and 3.

Any person who cannot be shown to have made a substantial contribution to the article cannot be listed as an author in the final version. The name of any person who is deemed to have made a minor contribution can, however, appear in the Acknowledgments section of the article.

Please complete the table below to indicate the contributions of all named authors to the manuscript.

Author Full Name:	Specification of Contribution to the Manuscript:
Yang Deng	design and perform most experiments, analyze data, and prepare the manuscript
Rui Duan	performed experiments and mice model construction
Wangli Ding	perform experiments and analyze the data
Qiuchen Gu	performed experiments
Manman Liu	mice model construction
Junshan Zhou	direct the study and prepare the manuscript
Jianguo Sun	direct the study and prepare the manuscript
Junrong Zhu	direct the study and prepare the manuscript

Please complete the table below to indicate the contributions of all named authors to the figures.

Figure 1:

R.J.Z., S.J.Z. and G.J.S. wrote the title of "OGD/R-ADEXs protects neurons against oxygen-glucose deprivation (OGD)/reoxygenation (R)", Y.D. performed experiments of Figure 1a-e, W.L.D. performed experiments of Figure 1f-g, Q.C.G. performed experiment of Figure 1h-i, Y.D. and W.L.D. analyzed the data.

Figure 2:

R.J.Z., S.J.Z. and G.J.S. wrote the title of "OGD/R-ADEXs protect neurons against oxygen-glucose deprivation/reoxygenation (OGD/R) injury through regulating autophagy", Y.D. performed experiments of Figure 2a-f and 2h-k, W.L.D. performed experiment of Figure 2g, Y.D. and W.L.D. analyzed the data.

Figure 3:

R.J.Z., S.J.Z. and G.J.S. wrote the title of "The regulation of autophagy by OGD/R-ADEXs depends on exosomes", Q.C.G. performed experiment of Figure 3a, W.L.D. performed experiment of Figure 3c, Y.D. performed experiments of Figure 3b and 3d-h, Y.D. and W.L.D. analyzed the data.

Figure 4:

R.J.Z., S.J.Z. and G.J.S. wrote the title of "OGD/R-ADEXs regulate autophagy and provide neuroprotection using Nampt", Q.C.G. performed experiments of Figure 4a-c, W.L.D. performed experiment of Figure 4d, Y.D. performed experiments of Figure 4e-i, Y.D. and W.L.D. analyzed the data.

Figure 5:

R.J.Z., S.J.Z. and G.J.S. wrote the title of "OGD/R-ADEX enhances autophagy through AMPK-mTOR signaling", W.L.D. performed experiments of Figure 5a-d, Y.D. and W.L.D. analyzed the data.

Figure 6:

R.J.Z., S.J.Z. and G.J.S. wrote the title of "OGD/R-ADEXs reduce brain injury and improve neurological function recovery after stroke", M.M.L. performed experiments of Figure 6a-b, R.D. performed experiments of Figure 6c-m, Y.D. and W.L.D. analyzed the data.

Signed for and on behalf of the Author(s):	Print Name:	Date:
Yang Deng, Rui Duan, Wangli Ding, Jianguo Sun, Junrong Zhu	Y.D., R.D., W.L.D., Q.C.G., M.M.L., S.J.Z., G.J.S., R.J.Z.	2022-11-07
